# Linking the Defective Structure of Boron-Doped Carbon Nano-Onions with Their Catalytic Properties: Experimental and Theoretical Studies

**DOI:** 10.1021/acsami.1c12126

**Published:** 2021-10-22

**Authors:** Grzegorz S. Szymanski, Yuka Suzuki, Tomonori Ohba, Bogdan Sulikowski, Kinga Góra-Marek, Karolina A. Tarach, Stanislaw Koter, Piotr Kowalczyk, Anna Ilnicka, Monika Zięba, Luis Echegoyen, Artur P. Terzyk, Marta E. Plonska-Brzezinska

**Affiliations:** † Faculty of Chemistry, Physicochemistry of Carbon Materials Research Group, Nicolaus Copernicus University in Torun, Gagarin Street 7, 87-100 Torun, Poland; ‡ Graduate School of Science, Chiba University, 1-33 Yayoi, Inage, 263-8522 Chiba, Japan; § Jerzy Haber Institute of Catalysis and Surface Chemistry, Polish Academy of Science, Niezapominajek 8, 30-239 Cracow, Poland; ∥ Faculty of Chemistry, Jagiellonian University in Kraków, Gronostajowa Street 2, 30-387 Kraków, Poland; ⊥ Faculty of Chemistry, Department of Physical Chemistry, Nicolaus Copernicus University in Torun, Gagarin Street 7, 87-100 Torun, Poland; # School of Engineering and Information Technology, Murdoch University, Murdoch, Western Australia 6150, Australia; ¶ Faculty of Chemistry, Nicolaus Copernicus University in Torun, Gagarin Street 7, 87-100 Torun, Poland; ∇ Department of Chemistry, University of Texas at El Paso, 500 W. University Avenue, El Paso, Texas 79968, United States; ○ Department of Organic Chemistry, Faculty of Pharmacy with the Division of Laboratory Medicine, Medical University of Bialystok, Mickiewicza 2A, 15-222 Bialystok, Poland

**Keywords:** carbon nanostructure, carbon nano-onion, defect, doping, boron, catalysis

## Abstract

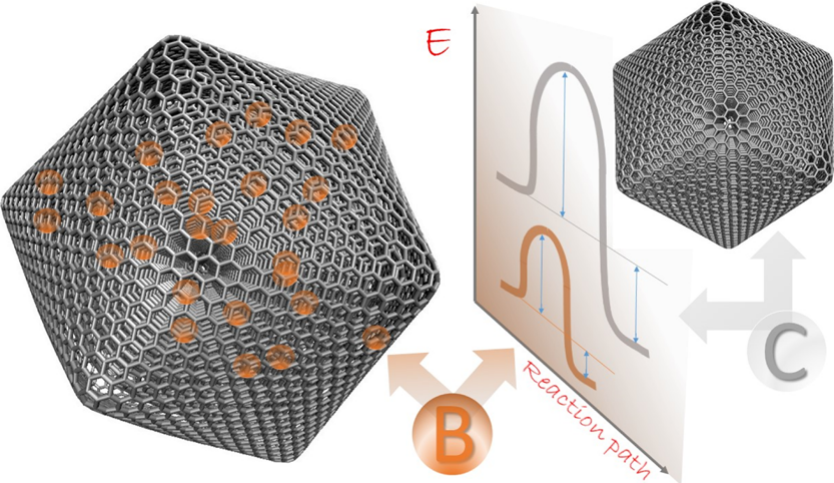

Defects are widely present in nanomaterials, and they are recognized as the active sites that tune surface properties in the local region for catalysis. Recently, the theory linking defect structures and catalytic properties of nanocatalysts has been most commonly described. In this study, we prepared boron-doped carbon nano-onions (B-CNOs) by applying an annealing treatment of ultradispersed nanodiamond particles and amorphous boron. These experimental conditions guarantee doping of CNOs with boron atoms in the entire carbon nanostructure, thereby ensuring structural homogeneity. In our research, we discuss the correlations between defective structures of B-CNOs with their catalytic properties toward SO_2_ and *tert*-butanol dehydration. We show that there is a close relationship between the catalytic properties of the B-CNOs and the experimental conditions for their formation. It is not only the mass of the substrates used for the formation of B-CNOs that is crucial, that is, the mass ratio of NDs to amorphous B, but also the process, including temperature and gas atmosphere. As it was expected, all B-CNOs demonstrated significant catalytic activity in HSO_3_^–^ oxidation. However, the subsequent annealing in an air atmosphere diminished their catalytic activity. Unfortunately, no direct relationship between the catalytic activity and the presence of heteroatoms on the B-CNO surface was observed. There was a linear dependence between catalytic activity and Raman reactivity factors for each of the B-CNO materials. In contrast to SO_2_ oxidation, the B-CNO-a samples showed higher catalytic activity in *tert*-butanol dehydration due to the presence of Brønsted and Lewis acid sites. The occurence of three types of boron-Lewis sites differing in electron donor properties was confirmed using quantitative infrared spectroscopic measurements of pyridine adsorption.

## 1 Introduction

The well-defined catalysts for efficient catalytic reactions has attracted considerable interest in recent decades. Although numerous efforts have been made to tailor the structure of carbon nanosystems toward catalysis,^1^ significant challenges remain to foster both their fundamental research and commercial applications. Recently, the theory linking defect structures and catalytic or electrocatalytic properties of nanocatalysts has been the most commonly described one.^2,3^ Defects are widely present in nanomaterials, and they are recognized as the active sites that influence catalytic properties of materials.^2,4^ With regard to the dimensions, defects in solid nanomaterials can be classified into four categories:^3,4^ (1) zero-dimensional point defects (e.g., doping, vacancy, and reconstruction), which contain nonmetallic atom doping-induced defects and metal defects;^2,5^ (2) one-dimensional line defects (e.g., dislocation); (3) two-dimensional planar defects (e.g., grain boundaries); and (4) three-dimensional volume defects (e.g., spatial lattice disorder). These defects may be created via *in situ* synthesis and by the application of post-modification methods; their location in the structure is, however, completely different. Such behavior greatly affects the electronic properties of nanomaterials and enables optimization of chemisorption of the key intermediates which, in turn, can trigger improved catalytic performance.^6^

Another important parameter known to influence catalytic properties is surface chemistry. When nonmetal dopants are introduced into the carbon layer, they may lead to the change of surface electronegativity between the heteroatom-doped and adjacent C atoms.^6^ Zheng and co-workers selected six nonmetallic heteroatoms with various electronegativities, which act as active sites for catalytic processes: N and O act as electron acceptors, and B, F, S and P act as electron donors.^7^ The heteroatom doping of the carbon nanostructures (CNs) is also recognized as carbon defects, which tailor the local charge in graphene layers and lead to the improvement of the catalytic effect, which is primarily influenced by the nature and number of the doped heteroatoms.^5^

It should be noted that not every atomic configuration is preferred. Density functional theory calculations indicated that for the B-doped CNs, the carbon π electrons are activated by conjugation with the vacant 2 p_*z*_ orbital of B. In these nanostructures, the B sites become active for the catalytic reaction.^8^ Apart from doping elements, other factors, such as the surface area/porosity/morphology of the materials, affect the catalytic activities.^6^ CNs easily form pores of different sizes (micro-, meso-, and macropores) owing to their flexible bonding and surface properties.

Our interest focuses on the catalytic and electrocatalytic processes that take place in a wide range of devices that may be based on CNs. Among all the candidates, graphene- and graphdiyne-based materials are perceived to be ideal for these processes due to their excellent conductivity and mechanical stability, large specific area with microporosity, easy production of defective motifs in their structures (e.g., pentagon defects, edge defects, and hole defects from micropores), and high carrier mobility.^8,9^ Carbon nano-onions (CNOs) belong to this family, consisting of quasi-spherical- and polygonal-shaped graphitic layers close to one another.^10^ The size, shape, and type of core are strongly affected by the preparation method of the onion-like structures. All CNOs exhibit a structure analogous to other CN graphene-based structures, in which each carbon atom is bound to three others and is sp^2^ hybridized. Each structure also contains some sp^3^ hybridized carbon atoms. Although the carbon atoms with sp^2^ hybridization are responsible for the chemical reactivity, the carbon atoms with sp^3^ hybridization are recognized as defective sites in CNs, which are primarily responsible for the catalytic efficiency of CNs. However, spherical CNOs with sizes less than 10 nm appear to be promising materials in catalysis and electrocatalysis, and the literature on this subject is still very scarce. The process of doping CNs was achieved by direct reactions during the preparation of these nanostructures (modification of the entire CNs) and by so-called post-modification (modification of the outer layer of graphene CNs only).^11^ Regardless of the method used, the reaction must lead to “incorporation” of boron atoms into the graphene layer of the CNs, thereby forming C–B–C or B–C–B moieties. In the brief discussion presented below, we did not include work and discuss the formation of onion-like carbons doped with metals. Our research is focused on analyzing the correlation between the catalytic effect and the nanocarbon structures.

In a few publications, we can find reports concerning the catalytic properties of pristine CNOs or their doped nanostructures. Pristine CNOs were used as a catalyst for styrene synthesis by oxidative dehydrogenation of ethylbenzene and the oxidative dehydrogenation of *n*-butane.^12^ The nitrogen-doped CNOs were used as efficient catalysts for epoxidation reactions,^13^ oxidative desulfurization^14^ and as electrocatalysts for oxygen reduction reaction (ORR),^15^ hydrogen peroxide and NO_2_^–^ determination.^16,17^ The codoped CNOs with nitrogen and boron or with nitrogen and sulfur were also used as electrocatalysts for ORR.^18,19^ The boron-doped CNOs (B-CNOs) showed interesting electronic performance and catalytic properties,^20,21^ for example, for reduction of nitroarenes.^22^ Although it was observed that pristine and heteroatom-doped CNOs have significant potential in catalysis and electrocatalysis, many issues remain unresolved.

Although numerous efforts have been made to tailor the structure of CNs toward catalysis, significant challenges still remain to impede their fundamental research. The presence of different types of defects in CNs exhibits significant difference in their catalytic properties. In this context, it is of major importance to develop a new group of the heteroatom-doped CNs with the controlled structural properties that affect their catalytic properties. In this study, we prepared B-CNO samples by applying an annealing treatment of ultradispersed nanodiamond (ND) particles in the presence of amorphous boron.^16,23^ These experimental conditions guarantee doping of the CNOs with boron atoms in the entire CN, thereby ensuring structural homogeneity. Here, we discuss the correlations between defective structures of B-CNOs with their catalytic properties toward SO_2_ oxidation and *tert*-butanol dehydration, which have not yet been studied.

## 2 Experimental Section

### 2.1 Preparation of B-CNOs

Commercially available ND powder with a crystal size between 4 and 6 nm (Carbodeon μDiamondMolto and ND content greater than 97 wt %) was used for the preparation of B-CNOs. Five hundred milligrams of NDs and 50 mg (or 25; or 10 mg) of boron were placed in a graphite crucible, and an annealing procedure was applied. The B-CNO nanostructures with the following mass ratio descriptors thus resulted: NDs to boron [*m*_NDs_/*m*_boron_ = 500/50 mg (10:1); *m*_NDs_/*m*_boron_ = 500/25 mg (20:1); *m*_NDs_/*m*_boron_ = 500/10 mg (50:1)], and these are labeled as the 1B-CNO, 2B-CNO, and 3B-CNO materials, respectively. The samples were prepared as described previously.^20^ One part of the subsequent B-CNO products (1B-CNO, 2B-CNO, and 3B-CNO) was additionally annealed in an air atmosphere at 450 °C. The materials that were additionally annealed are defined as 1B-CNO-a, 2B-CNO-a, and 3B-CNO-a.

### 2.2 Methods

High-resolution transmission electron microscopy (HRTEM) was performed using a Titan G2 HRTEM microscope operated at 300 kV (FEI company), equipped with a field emission gun. HRTEM imaging of the sample microstructure was performed in bright field mode using a charge-coupled device (CCD) camera as a detector. Sample mapping, determination of the distribution (distribution of elements in the samples) was performed in the scanning TEM (STEM) mode, collecting the energy dispersive spectroscopy (EDS) spectrum from each place corresponding to the map pixels, point by point. The powder materials were prepared by their dispersion in ethanol. Next, a dispersion drop was placed on a lacey-carbon grid (200 mesh), and the solvent was evaporated.

^11^B solid-state MAS NMR spectra were acquired at 160.47 MHz on a Bruker Advance III 500 MHz WB spectrometer (11.7 T). The samples were spun in zirconia rotors at 10 kHz. Inversion recovery measurements carried out on the chosen samples led to an estimation of the *T*_1_ relaxation times in the range of 13.5–30 ms. ^11^B MAS NMR spectra were recorded with parameters: 0.4 μs (single-pulse excitations), 250 kHz, 1 s (repetition time) and 10 240 transients.^24^^11^B chemical shifts are reported in ppm from an external 1 M boric acid solution. Deconvolutions of the spectra were carried out using the Bruker TopSpin 3.1 software.

X-ray photoelectron spectroscopy (XPS) was performed using an ultrahigh vacuum chamber (PREVAC) (base pressure below 10^–8^ mbar) using a nonmonochromatic Al Kα (1486.7 eV; 12 kV; 30 mA) radiation source (VG Scienta SAX 100) and monochromator (VG Scienta XM 780). The emitted photoelectrons were detected using a Scienta R4000 hemispherical analyzer. For all samples, a low-resolution survey run (0–1350 eV) at a pass energy of 200 eV was performed. The C 1s, B 1s, O 1s, and N 1s high-resolution spectra were recorded at a pass energy of 20 eV and at room temperature. The C 1s, O 1s, B 1s, and N 1s spectra were fitted by the Gaussian–Lorentzian functions after the Shirley background subtraction. The peaks were fitted using the CasaXPS software (Casa Software Ltd.). The C KLL spectra X-ray induced Auger electron spectroscopy (XAES) were taken from XPS. The first derivative XAES spectra were obtained using a 25-point Savitzky–Golay quadratic polynomial differentiation method.

The room-temperature Raman spectra were investigated with a Bruker Optik Senterra confocal spectrometer equipped with a thermoelectric cooled CCD detector. The parameters used for the Raman measurements: a laser with a wavelength of 532 nm (2.33 eV); 2 mW (power of the laser beam), and 2 cm^–1^ (the spectral resolution). The spectra obtained after normalization were deconvoluted using the Voigt functions in the OriginPro 7.5 software (OriginLab Corp.).

The X-ray diffraction analysis (X′Pert Pro-diffractometer with X′Celerator Scientific detector, Panalytical) was performed using a Cu Kα 1.54 Å anode, in the angular range 10–100° (2θ), step: 0.016° (2θ) (exposure time per step 60 s), and scanning speed: 0.03 °/s, on an amorphous glass as a substrate.

Nitrogen adsorption–desorption isotherms (77 K) were measured using an ASAP 2020 Plus 2.00A (Micromeritics, USA) adsorption apparatus. Water vapor adsorption isotherms were measured at 303 K using a volumetric apparatus (BELSORP-max, MicrotracBEL Co., Osaka, Japan). Before adsorption–desorption measurements, all samples were subjected to vacuum evacuation at 473 K for more than 2 h.

The quantitative infrared (IR) spectroscopic studies of Py-adsorption were conducted in the transmission mode. The catalysts were mixed with SiO_2_ in a 1:3 ratio and pressed into a thin self-supported disk and placed in the custom made quartz cell. Each sample was treated under vacuum at a temperature of 350 °C for 30 min in order to remove all the preadsorbed impurities. Then, the IR cell was cooled to 45 °C and the catalyst was saturated with pyridine (Py) vapors followed by the desorption at the same temperature to allow for removal of the gas and the physically adsorbed Py molecules. The spectra (1000 scans per spectrum) were recorded with a resolution of 2 cm^–1^ using a Bruker Vertex 70 spectrometer. The spectra were normalized to 10 mg catalyst.

### 2.3 Measurements of Catalytic Activity

The decomposition of *tert*-butanol by B-CNOs was investigated in a fixed bed flow-type microreactor by the micropulse technique.^25^ The product analyses were performed by on-line gas chromatography. The catalytic tests were conducted in the temperature range of 393–523 K at 10 degree increments. At a given temperature, 1 μL of the reactant was injected into the reactor. In addition, the catalytic activity of B-CNO in SO_2_ oxidation was measured at 303 K using a batch reactor in SO_2_ solution (1.65 × 10^–3^ M SO_2_) and constant O_2_ concentrations ([O_2_] > 10^–3^ M) with continuous air bubbling inside the reactor (3 dm^3^/h). The SO_2_ conversion was monitored conductometrically.^26,27^

## 3 Results and Discussion

### 3.1 Microscopic and Structural Analysis

**Figure 1:**
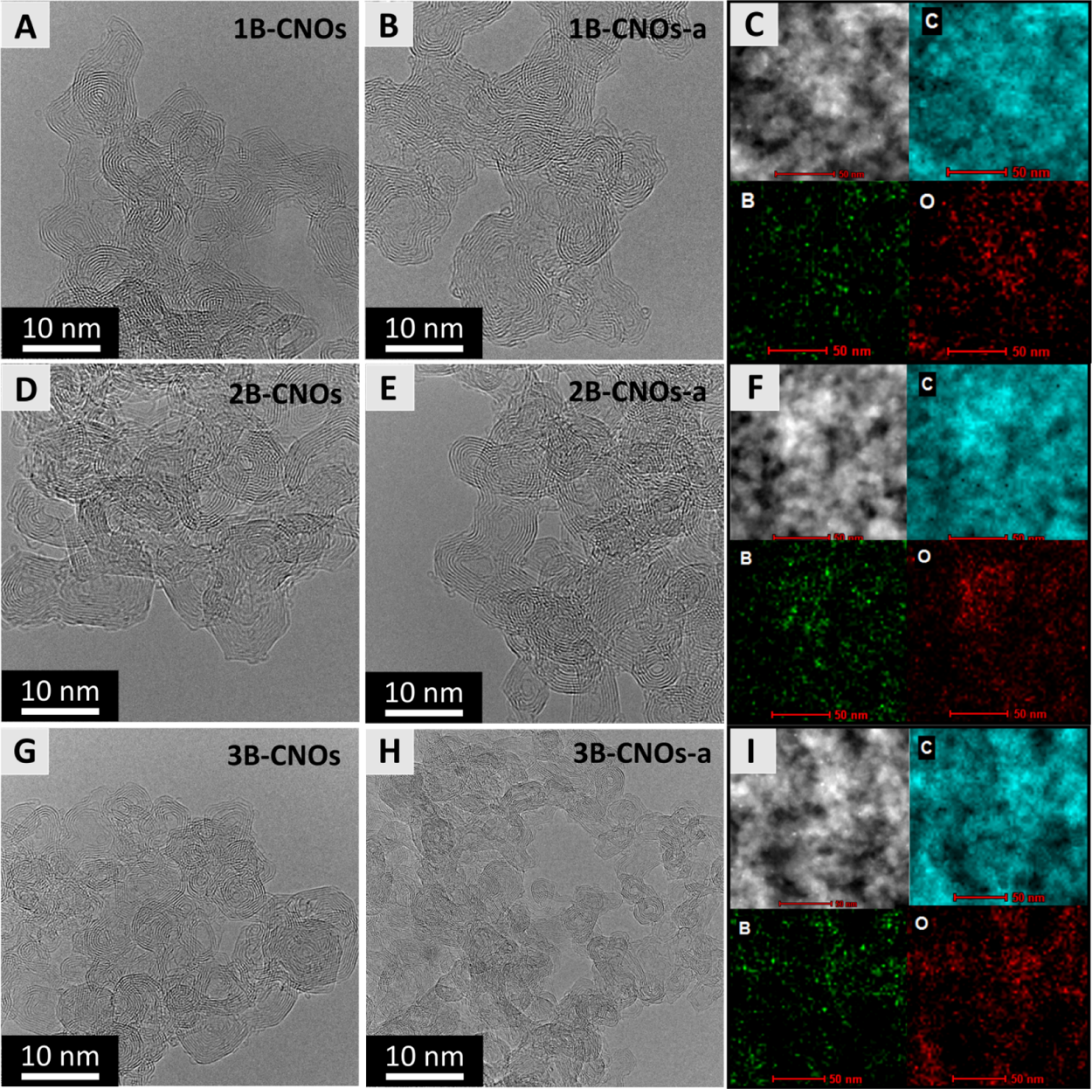
(A,B,D,E,G,H) HRTEM images with (C,F,I) EDS analysis of the (A) 1B-CNOs, (B,C) 1B-CNOs-a, (D) 2B-CNOs, (E,F) 2B-CNOs-a, (G) 3B-CNOs and (H,I) 3B-CNOs-a.

In this study, we used the modified Kuznetsov method for the preparation of B-CNOs by applying an annealing treatment under an inert atmosphere and reduced pressure of ultradispersed ND particles and amorphous boron.^16,23^ Using this procedure, polygonal dense-core CNOs were formed with a diameter of ca. 6–10 nm under all experimental conditions with no significant changes for different component mass ratios. The only difference seen with HRTEM images is that the nanostructures subjected to additional annealing treatment do not have an amorphous carbon layer on the surface of the B-CNOs (Figures 1 and S1–S3). Sample mapping and determination of the distribution of elements in the samples (C, B, O, and N) was performed in the STEM mode, collecting the EDS spectrum from each place corresponding to the map pixels, point by point (Figures 1 and S1–S3). The collected maps are presented in the form of a matrix of pixels, with different colors for the mapped elements and the intensities corresponding to the percentages of the elements. The EDS analysis confirmed that the highest content of B was observed for the 1B-CNOs and 1B-CNOs-a, where the highest content of amorphous boron was used for the preparation of the B-doped CNOs from the NDs (Figures 1C,F,I and S1–S3).

The XPS measurements indicate that despite different starting amorphous B to ND ratios, all B-CNOs exhibit quite similar elemental surface compositions (Table 1). In addition to B being present as a heteroatom, all samples also contain some amount of O and N on the surface. The presence of N in all B-CNO samples, results from the NDs, where it is typically found as an impurity.^28^ N was not detected in the undoped CNOs-a.^11^ This suggests that N is included in the C lattice in association with B, as reported for other co-doped carbon materials.^29,30^ B stabilizes N in the hexagonal C lattice due to great affinity between B and N.^28,29^ The influence of B on the N presence in carbon materials was also observed earlier by Camisasca et al. for BN-CNOs prepared in similar way.^28^

**Table 1:** Surface Elemental Composition, D and sp^2^ C % Values of B-CNOs Determined by XPS and XAES, Respectively

	elements (at. %)					sp^2^ C (%)	
sample	C	O	N	B	*D* (eV)	XPS	XAES
1B-CNOs	90.5	2.2	3.3	4.0	19	90.3	58.6
2B-CNOs	92.0	2.4	1.9	3.7	19	90.7	58.6
3B-CNOs	94.2	1.2	2.1	2.6	20.5	92.1	73.7
1B-CNOs-a	91.4	2.1	2.9	3.7	20	92.2	68.7
2B-CNOs-a	91.4	2.4	2.3	4.0	19.5	90.1	63.6
3B-CNOs-a	93.7	1.7	1.7	3.0	21	92.3	78.8
CNOs-a^a^	98.0	2.0					

a Data from ref (11).

**Figure 2:**
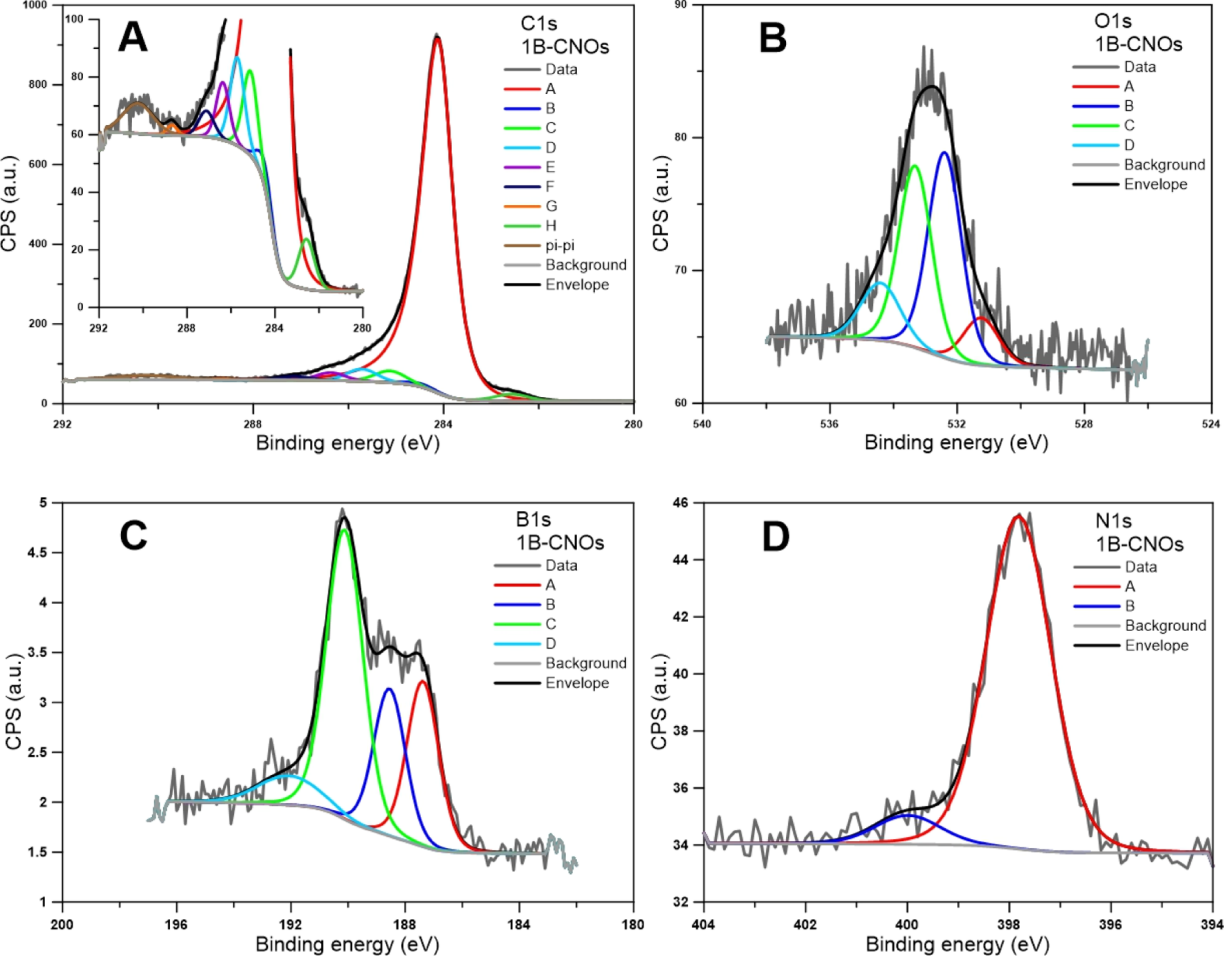
XPS spectra of the 1B-CNOs: (A) C 1s, (B) O 1s, (C) B 1s and (D) N 1s spectral regions.

The total concentration of heteroatoms in the B-CNOs increases slightly upon increasing the starting B to ND ratio (Table 1). Whereas subsequent annealing of B-CNOs at 450 °C in an air atmosphere did not notably change the total amount of doped heteroatoms, it did change the distribution of heteroatom species. The type and quantity of the B-CNO surface functionalities containing O, B and N atoms were obtained from the deconvolution of the high-resolution spectral regions, which is presented in Figure 2 for 1B-CNOs (C 1s, O 1s, N 1s and B 1s). The assignments and the percentages of each component derived from the spectral deconvolutions are summarized in Table 2. The details of curve fitting are given in the Supporting Information (Tables S1–S3). Based on the deconvolution of XPS C 1s spectra for all samples, phenol, ether, carbonyl and carboxyl/lactone groups, boron carbide species, and sp^2^-hybridized C atoms in strained C=C bonds due to the curvature of the CNOs were identified (Figure 2A and Table 2). The deconvoluted XPS O 1s regions indicate the presence of O–B bonds (∼531.5 eV) and confirm the formation of carbonyl (∼531.5 eV), phenol (∼532.5 eV), ether (∼533.4 eV), and carboxyl/lactone species (∼534.5 eV) during the formation of B-doped CNs (Figure 2B and Table 2). The B 1s XP spectra can be resolved into four regions, which correspond to B atom clusters [B^0^ (187.4 eV)], different B carbide species [B_4_C (∼187.4 eV), BC_3_ (∼188.5 eV)], various forms of oxidized B [B_6_O (∼187.4 eV), BC_2_O (∼190.1 eV), B_2_O_3_ (∼192.1 eV)] and B–N bonds (∼190.1 eV, originating from the chemical bonding between the inherent trace N species and B after annealing) (Figure 2C and Table 2). The concentration of the oxidized B forms (B_2_O_3_) increased after annealing in an air atmosphere as a result of oxidation of some B and B carbide species, which probably remained in excess after the CNO formation process. The XPS N 1s regions were fitted with two peaks corresponding to N–B (397.8 eV) and C–N (399.4 eV) bonds in boron nitride and amine species (Figure 2D and Table 2). Boron nitride species are the most abundant forms of N, in contrast, to previously reported studies for BN-CNOs, where pyridinic species prevail.^28^

**Table 2:** Distribution of Elements Obtained from the Deconvolution of the C 1s, O 1s, B 1s and N 1s Spectra by XPS

					concentration (at. %)					
region	peak	BE (eV)	assignment	references	1B-CNOs	2B-CNOs	3B-CNOs	1B-CNOs-a	2B-CNOs-a	3B-CNOs-a
C 1s	A	284.2 ± 0.1	C–C sp^2^	(20, 81)	81.05	83.05	86.45	84.14	80.25	86.35
	B	284.6 ± 0.1	strained C–C sp^2^	(81)	0.63	0.43	0.30	0.12	2.07	0.11
	C	285.1 ± 0.1	C–C sp^3^	(20, 81)	2.45	2.86	2.64	1.75	3.56	1.91
	D	285.7 ± 0.1	C–OH/C=N	(79−81)	2.42	2.33	2.07	1.87	2.59	2.34
	E	286.4 ± 0.1	C–O–C/C–N	(81, 82)	1.43	1.14	0.97	1.20	1.10	1.36
	F	287.1 ± 0.1	C=O	(81, 82)	0.76	0.57	0.62	0.51	0.23	0.32
	G	288.7 ± 0.1	O=C–O–R	(80)	0.19	0.14	0.00	0.00	0.09	0.20
	H	282.6 ± 0.1	C–B	(20, 28)	1.52	1.53	1.15	1.79	1.48	1.11
O 1s	A	531.5 ± 0.1	O–B/C=O	(22, 81)	0.22	0.25	0.15	0.34	0.31	0.30
	B	532.5 ± 0.1	C–OH/epoxy	(22, 81)	0.90	1.02	0.46	0.83	0.90	0.71
	C	533.4 ± 0.1	C–O–C	(81, 83)	0.79	0.90	0.48	0.72	1.00	0.59
	D	534.5 ± 0.1	H_2_O, R–O–C=O	(83)	0.30	0.20	0.10	0.19	0.20	0.09
B 1s	A	187.3 ± 0.1	B^0^, B_4_C, B_6_O	(20, 22, 74)	0.94	0.82	0.67	0.86	0.79	0.67
	B	188.5 ± 0.1	B–C, BC_3_	(20, 22, 28, 74)	0.84	0.68	0.67	0.75	0.78	0.55
	C	190.1 ± 0.1	BC_2_O, B–N	(20, 22, 74)	1.89	1.85	0.95	1.54	1.90	1.22
	D	192.1 ± 0.1	B_2_O_3_	(20, 22, 28, 74)	0.32	0.35	0.31	0.55	0.53	0.56
N 1s	A	397.8 ± 0.1	N–B	(28, 71)	3.05	1.75	1.84	2.63	2.07	1.42
	B	399.4 ± 0.1	N–C	(71, 86)	0.26	0.10	0.22	0.25	0.19	0.25

The typical XPS analysis often overlooks the X-ray-generated Auger characteristics. In the case of the C atom, this feature is present at about 265 eV (kinetic energy, KE) and it is due to the relaxation followed by the ejection of valence electrons from the C atom after the initial X-ray photoionization (C KLL). The shape of the C KLL feature varies with the location of C atoms on the analyzed surface.^31,32^ These changes are explained by differentiation of the C KLL spectra. The energy difference between the maximum and minimum of the resultant curves changes significantly from sp^2^- to sp^3^-hybridized C atoms. This difference is usually designated as the *D* parameter. There exists a linear correlation between the measured *D* parameter and the estimated percentage of sp^2^-hybridized C atoms in the nanostructures.^32^ The *D* parameter is estimated for model samples, that is, for diamond and graphite, these evaluated values are 13.2 and 23.1 eV, respectively.^32^ The estimated values of the *D* parameter and the content of sp^2^-hybridized C atoms for B-CNOs are shown in Table 1. The sp^2^ C amount established from the C KLL analysis is regularly lower than that determined by the C 1s peak analysis. This divergence can be explained by the difference in the mean free path or escape depth of the electrons adequate to each peak. As the KE of C KLL Auger electrons (272 eV), is lower than that of C 1s photoelectrons; therefore, the escape depth of the Auger electrons is smaller.^32,33^ Thus, the lower value for the sp^2^-hybridized C atom content obtained from the XAES analysis, in comparison, to the results of the XPS C 1s analysis can be elucidated by the presence of sp^3^-hybridized C atoms on the surface of the B-CNOs. Thermal treatment in an air atmosphere at 450 °C increases the sp^2^-hybridized C atom content in the annealed B-CNO materials (Table 1).

^11^B nuclei are quadrupolar with a corresponding spin equal to 3/2. The quadrupolar moment of the ^11^B nuclei is two times lower than that of ^10^B, and this property coupled with the natural abundance of 80%, gives rise to the relatively high sensitivity of ^11^B NMR measurements. Therefore, ^11^B NMR spectroscopy is broadly used for monitoring the behavior and evolution of B nuclei in a number of materials, exemplified by zeolites, mixed oxides, glasses and a plethora of other B-containing samples.^34,35^

**Figure 3:**
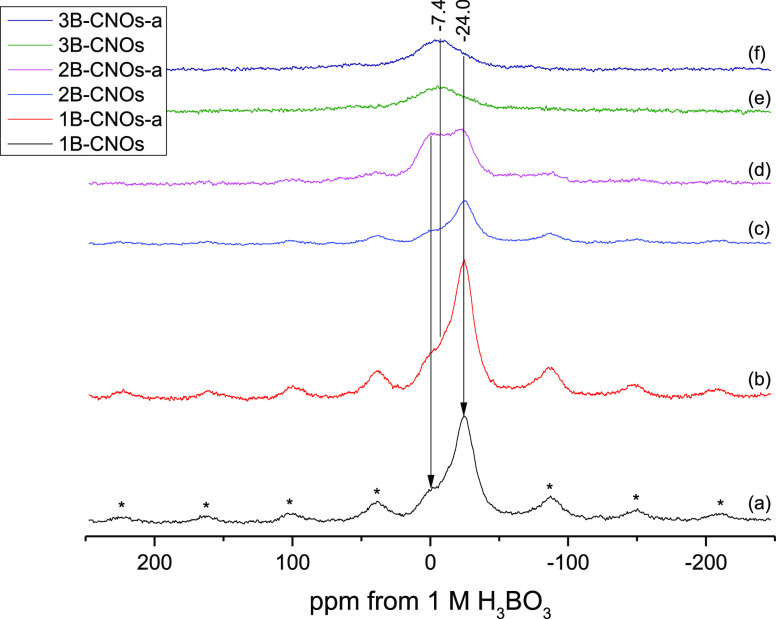
Comparison of the ^11^B MAS NMR spectra of (A) 1B-CNOs; (B) 1B-CNOs-a; (C) 2B-CNOs; (D) 2B-CNOs-a; (E) 3B-CNOs, and (F) 3B-CNOs-a acquired at 160.5 MHz and a magic-angle-spinning speed of 10 kHz. The asterisks denote spinning side-bands.

The ^11^B MAS NMR spectra are shown in Figure 3. The 1B-CNO, 1B-CNO-a, 2B-CNO, and 2B-CNO-a samples exhibit similar features, with strong signals centered at −24 ppm and weak signals centered at ca. 0 ppm. Closer inspection of the spectra (A) to (D) also reveals the presence of shoulders situated between them. These are also many spinning sidebands. Taking into account the general features found in the spectra of various B-containing materials, the signal at −24 ppm can be assigned to four-coordinate boron atoms. The characteristic behavior found previously in ^10^B MAS NMR spectra of other CNOs points to the presence of trigonal boron species at ca. 0 ppm.^20^ These species reveal a generally higher quadrupolar coupling constant (*C*_Q_) in comparison with the four-coordinate B signals found at −24 ppm (Table 3). Finally, weaker signals at approximately −11 ppm may be assigned to distorted tetrahedral B species.

**Table 3:** Chemical Shift (σ_B_), Quadrupolar Coupling Constant (*C*_Q_) and Concentration (at. %) of Boron Sites as Derived from the ^11^B MAS NMR Spectra Visualized in Figure 3

sample	signal	chemical shifts σ_B_ (ppm)	*C*_Q_^a^ (kHz)	η^b^	concentration (at. %)	*R*^c^ (%)
1B-CNOs	1	0.2	125	0	8.5	
	2	–11.5	204	0	10.6	96.44
	3	–24.9	93	0.09	80.9	
1B-CNOs-a	1	0.7	126	0	12.0	
	2	–12.2	126	0.01	19.4	96.47
	3	–24.7	96	0	68.6	
2B-CNOs	1	0.4	207	0.08	9.7	
	2	–12.8	131	0	10.3	96.04
	3	–25.0	93	0.07	80.0	
2B-CNOs-a	1	2.2	126	0.02	30.3	
	2	–10.1	126	0.08	26.5	96.85
	3	–23.8	106	0.01	43.2	
3B-CNOs	1	–5.5	126	0	100	94.00
3B-CNOs-a	1					
	2	–7.3	126	0	100	92.75
	3					

a Quadrupole coupling constant.

b Asymmetry parameter.

c Correlation coefficient.

Deconvolutions of the spectra were performed considering the central signals and first-order spinning side bands, and the fitting results are shown in Table 3. Correlation coefficients were better than 96%, excluding the 3B-CNO samples with one broad signal and slightly lower *R* parameter. Estimation of quadrupolar coupling constants and asymmetry parameters were also conducted. Representative examples of deconvolution are shown in the Supporting Information (Figures S4 and S5). The four spectra of the 1B-CNO and 2B-CNO samples (Figure 3A–D) exhibit similar characteristics. The number of tetrahedrally coordinated boron atoms at −24 ppm predominate. Upon annealing in air, the intensities of signals 1 and 2 increase at the expense of 3; thus, more distorted tetrahedral and trigonal species are produced during the process. The changes are, however, not monotonic in character.

The spectra for 3B-CNOs and 3B-CNOs-a are nearly identical (Figure 3E,F)—only one broad signal located at an intermediate position (−6, −7 ppm) is observed. It is difficult to establish whether the signal is due to a single boron species with a distorted tetrahedral coordination or the overlap of several signals. Another possibility is that trigonal and tetragonal signals present in the 1B- and 2B-CNO samples could coalesce in the 3B-CNO samples, which could be due to their lower crystallinity and/or higher disorder. On the other hand, the signals in the range from −5.7 to −6 ppm could indicate the presence of boron carbide B_4_C formed during sample preparation. For example, a sample annealed at 2000 °C gave a broad ^11^B signal, with a chemical shift of −6 ppm.^36^ Finally, the asymmetry parameters for 3B-CNO are equal to 0, and the quadrupole coupling constants are very low (0.13 MHz Table 3). Reported in literature *C*_Q_ values for B_4_C range from 0.1 to 0.7 MHz.^37,38^

**Figure 4:**
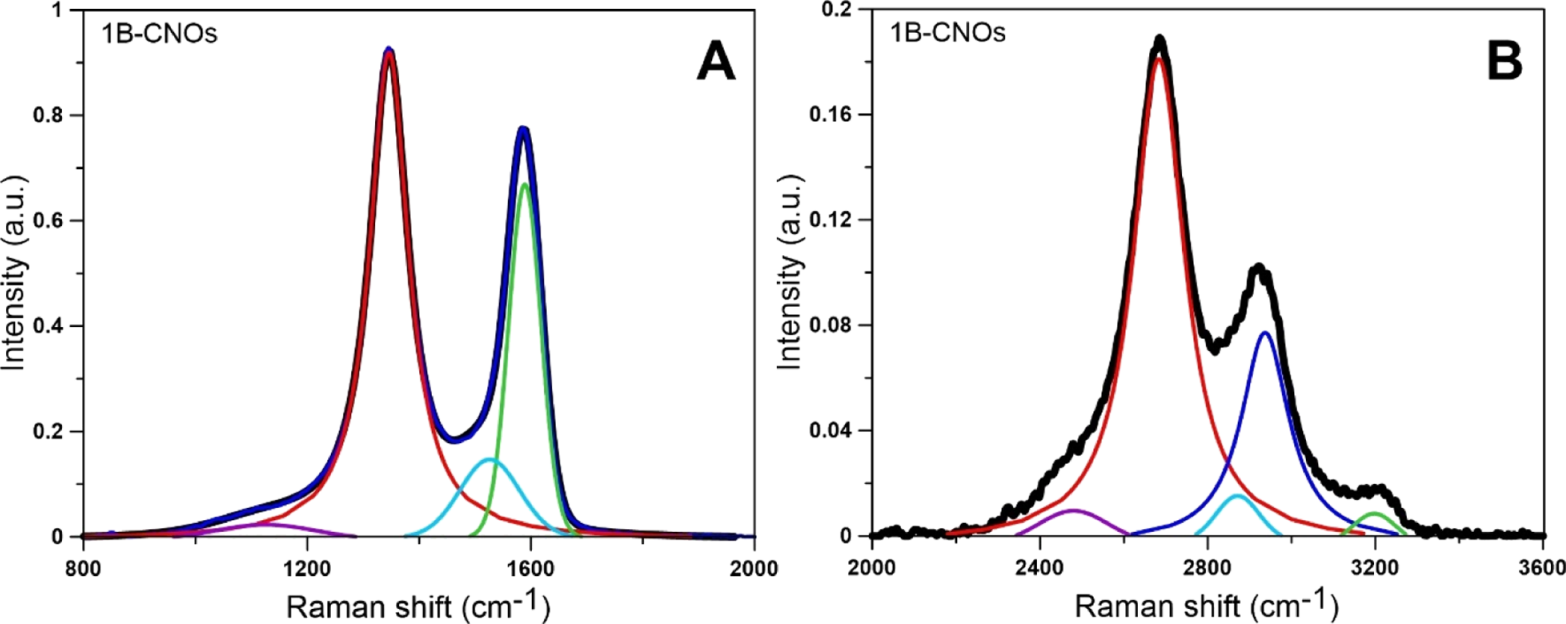
Decompositions of Raman spectra of first-order (A) and second-order (B) 1B-CNOs on several spectral components (λ = 532 nm). Please see the text and Table S4 for details.

The Raman spectra of the 1B-CNOs were recorded in the 800–3600 cm^–1^ region, as illustrated in Figure 4. As shown, the curves exhibit three broad and overlapping peaks located at 1348, 1586, and 2700 cm^–1^, respectively. The Raman spectra of carbon materials possess usually two characteristic regions. The first-order region with the band at approximately 1580 cm^–1^ (G band) is attributed to the E_2g_ stretching vibration mode for the graphitic layers.^39−41^ In the case of highly disordered carbons, additional bands appear due to the presence of defects in the microcrystalline lattices. They are located at approximately 1350 cm^–1^ (D1), 1620 cm^–1^ (D2), 1500 cm^–1^ (D3), and 1200 cm^–1^ (D4), respectively.^39−41^ The D1 and D2 bands were assigned to the stretching vibrations of the disordered graphitic lattice. D3 was related with the amorphous sp^2^-bonded C atoms, which are responsible for the chemical and catalytic reactivity. D4 corresponds to sp^3^ or sp^2^–sp^3^ bonding with oxygen groups, which may be associated to the active sites on the carbon surface. The 1530 cm^–1^ band is very broad in the range of 1500–1550 cm^–1^, which results from the presence of organic molecules and fragments of functional groups.^42,43^ This finding may also be related to the reactive sites in the materials and consequently, to their reactivity. The band at 1200 cm^–1^ arises in very disordered carbon materials, such as soot and coal chars.^40,43^ Its origin is still controversial. In some cases, it was attributed to the presence of polyene-like structures or sp^3^–sp^2^ mixed sites at the periphery of crystallites, which may also be responsible for the reactivity of materials.^41^

Bands in the second-order region (2000–3600 cm^–1^) are caused by the overtone and combination of the first-order lattice vibration modes.^37^ The band at 2670 cm^–1^ is ascribed to an overtone of the D1 band (2D1). The combination of the G and D1 bands (G + D1) results in the band at 2920 cm^–1^. The 3180 cm^–1^ band is assigned to the overtone of the G band (2G).^40,44,45^ A band at approximately 2480 cm^–1^ is observed in the Raman spectra of the B-CNOs as a result of the combination of the D1 and D4 bands. The band at approximately 2700 cm^–1^ (2D band) can be assigned to the ordered graphitic structure, more sensitive to the change in structural disorder.^40^ The 2D1 band was broadened and shifted to lower wavenumbers and even disappeared when the CNs became less ordered.^43,46^ The intensities (band area) of the G and 2D1 bands in the Raman spectra were attributed to the structural disorder of the CNs.^43^

To enable detailed analysis of the Raman results, the spectrum was deconvoluted into four bands (Voigt type) in the first-order region and into five bands in the second-order region (Figure 4, Tables S4 and S5). As the FWHM cannot be reflected in the peak intensity ratio^47,48^ and the D and G peaks of the Raman spectrum are the sum of D1, D3, D4, and G peaks,^47^ the peak area ratios (*A*_D1_/*A*_G_) and (*A*_D3_/*A*_G_) were used to establish the order of the B-CNO crystalline structure in the present study (Table 4).^41,47^ In addition, *A*_D3_/*A*_G+D3_, *A*_D3_/*A*_G+D3_, and *A*_D3+D4_/*A*_G_ were determined and are gathered in Table 4. Seong and Boehman found^49^ that the *A*_D3_/*A*_G_ and *A*_D3_/*A*_G+D3_ ratios are good parameters to indicate the abundance of amorphous carbon. A higher value of (*A*_D3+D4_)/*A*_G_ and a lower value of *A*_G_/*A*_all_ may be attributed to the superior carbon combustion reactivity.^41,43^ The carbon oxidative reactivity is exactly related to the amount of edge sites from *A*_D1_/*A*_G_ and amorphous carbon from *A*_D3_.^48^ The Raman parameters are summarized in Table 4.

**Table 4:** Raman Parameters of B-CNOs

parameter	1B-CNOs	2B-CNOs	3B-CNOs	1B-CNOs-a	2B-CNOs-a	3B-CNOs-a	CNOs-a
ν_D1_ (cm^–1^)	1346	1338	1338	1340	1338	1336	1346.2
D1 FWHM (cm^–1^)	85	90	83	92	89	83	76.0
ν_G_ (cm^–1^)	1589	1578	1580	1581	1581	1580	1591.6
G FWHM (cm^–1^)	69	70	70	69	69	69	69.9
*A*_D1_/*A*_G_	2.32	2.45	2.22	2.66	2.63	2.47	1.860
*A*_D3_/*A*_G_	0.44	0.53	0.41	0.58	0.63	0.60	0.144
*A*_D4_/*A*_G_	0.13	0.19	0.16	0.19	0.18	0.20	0.041
*A*_D3_/*A*_G+D3_	0.31	0.35	0.25	0.37	0.40	0.39	0.126
*A*_D3+D4_/*A*_G_	0.58	0.72	0.57	0.76	0.81	0.79	0.185
*A*_G_/*A*_all_	0.26	0.24	0.264	0.23	0.23	0.24	0.328
*A*_Dtot_/*A*_G_	2.90	3.16	2.789	3.42	3.44	3.27	2.045
*A*_2D1_/*A*_G_	0.81	0.63	0.84	0.72	0.83	0.75	0.429
*L*_a_ (nm)	8.3	7.8	8.7	7.2	7.3	7.8	10.3

There are only small differences in the calculated parameters for all B-CNOs despite the different ratios of starting materials used during their preparation (NDs to amorphous B). The highest parameter values were observed for 2B-CNOs (*m*_ND_/*m*_B_ = 20:1). After additional annealing in an air atmosphere of B-CNOs (B-CNOs-a), increasing *A*_D1_/*A*_G_, *A*_D3_/*A*_G_, *A*_D3_/*A*_G+D3_, *A*_D3+D4_/*A*_G_ and *A*_D4_/*A*_G_ band ratios are observed for all annealed materials. This finding suggests that different additional structural defects and the carbon crystallites imperfections were generated during additional heat treatment in an air atmosphere. It is generally accepted that microcrystalline planar size is inversely proportional to the *A*_D1_/*A*_G_ ratio.^41,43^ Therefore, the increase in the *A*_D1_/*A*_G_ ratio implies a decline in the average planar size of the graphitic microcrystallites (*L*_a_) (Table 4).

The values of *L*_a_ gathered in Table 4 were calculated from *A*_D1_/*A*_G_ using eq 1 postulated by Cançado et al.^50^
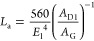
1where *E*_l_ is the laser energy (eV), *A*_D_/*A*_G_ is the area ratio of D1 to G, and *E*_l_ is 2.3 eV for 532 nm. For B-CNOs, the average planar size of the graphitic microcrystallites (*L*_a_) varies in the range of 7.2–8.7 nm, which is in good agreement with HRTEM studies (Figures 1 and S1–S3).

**Figure 5:**
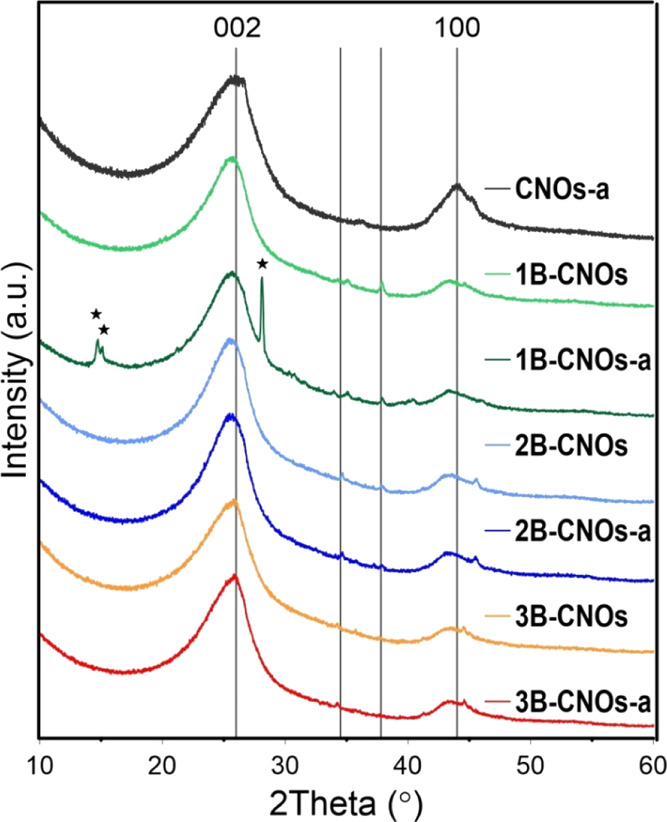
XRD patterns of B-doped CNOs.

X-ray diffraction (XRD) patterns of undoped CNOs and B-doped CNOs are shown in Figure 5, and they are similar to results previously reported.^20^ For all patterns, a broad reflection in the range between 23 and 27° and a second one with a maximum at 43.0° were observed.^51^ The broad signal in the range between 23 and 27° indicates the presence of undoped and B-doped graphite layers and their structural defects. Additionally, the signal broadening may result from differently sized nanoparticles within the sample. Diffraction profiles of the 1B-CNO and 2B-CNO samples revealed the presence of small peaks at 35.1 and 37.8° that correspond to a graphitic phase and boron carbide.^52,53^ Finally, the three sharp peaks at 14.6, 14.8 and 27.7° in the diffraction pattern of the 1B-CNOs-a sample correspond to 002, 010, and 100 indices of crystalline boric acid.^54^

### 3.2 Porosity Studies and Water Adsorption

Nitrogen adsorption–desorption isotherms are of type II and show the H3 type of IUPAC hysteresis, which means that pores are formed between CNOs (so-called external porosity). There were no remarkable differences in the pore size distributions between the studied samples and pore diameters were in the range of 6–20 nm (Figure S6). The heat treatment in an air atmosphere led to a slight increase in the Brunauer–Emmett–Teller (BET) surface area by several m^2^/g (Table 5).

**Table 5:** Fitted Parameters of Eq 2 from Water Adsorption Isotherms

sample	*S*_BET_ (m^2^/g)^a^	*R*^2^	*a*_0L_ (mg/g)	*a*_0L_ (mg/m^2^)	*K*_L_	*a*_0_ (mg/g)	*c*
1B-CNOs	206	0.9999	5.34	25.97	3.992	1.89	0.971
2B-CNOs	253	0.9998	3.64	14.37	3.700	2.016	0.979
3B-CNOs	288	0.9997	1.6	5.55	4.225	2.21	1.003
1B-CNOs-a	220	0.9995	83.8	381	21.038	16.37	0.632
2B-CNOs-a	266	0.9999	5.86	22.01	3.655	2.86	0.978
3B-CNOs-a	294	0.9999	2.79	9.49	14.91	15.35	0.641

**Figure 6:**
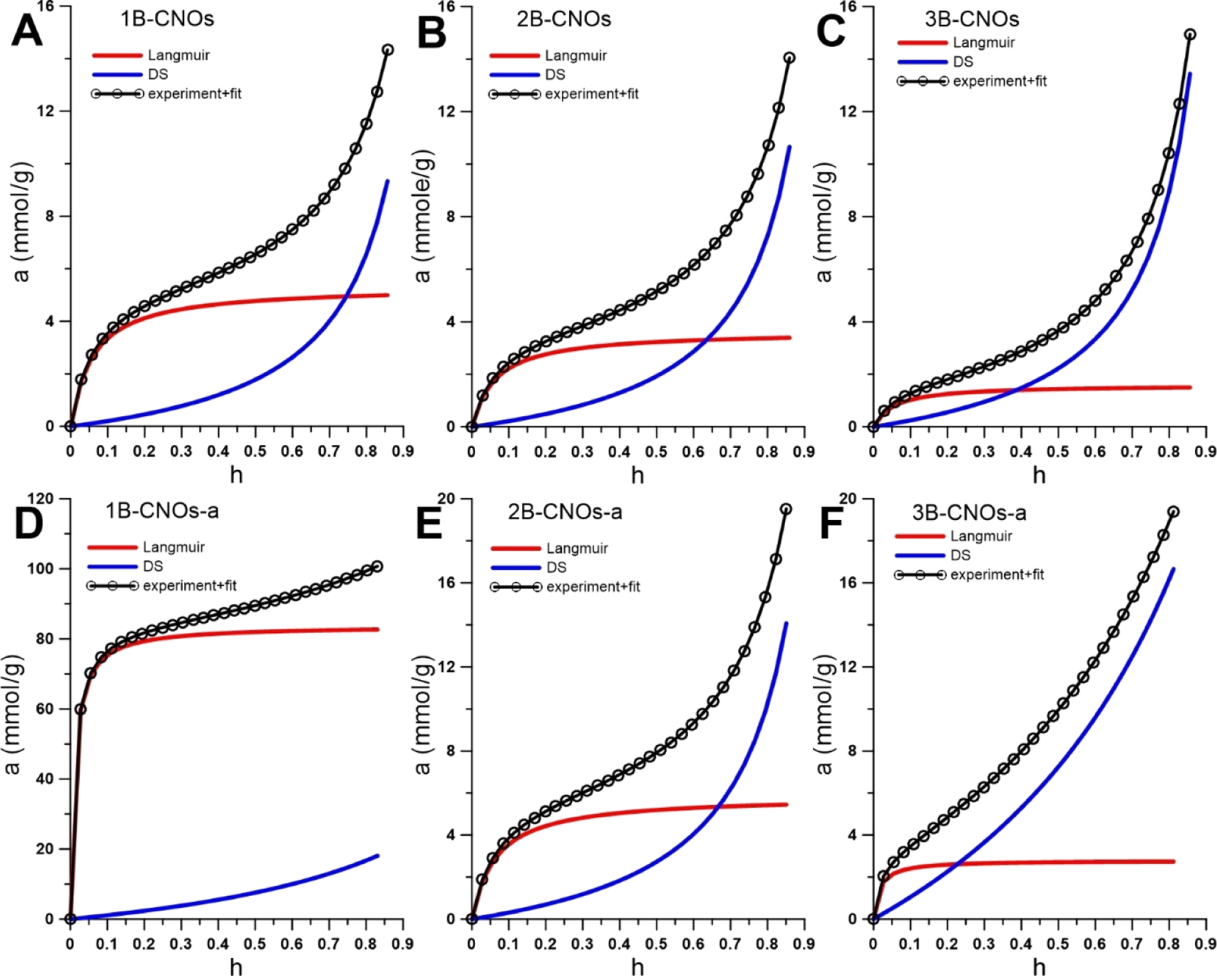
Water adsorption isotherms (symbols) at 303 K for the studied nanomaterials. Solid line shows the fitting using eq 2 with the separated adsorption from the Langmuir (red solid line) and Dubinin–Serpinski (blue solid line) contributions.

Water adsorption–desorption isotherms (type IV) show that the B-CNO surface is hydrophilic (Figure 6). To fit the water adsorption isotherms, the model proposed by D’Arcy and Watt was used.^55^ This model assumes that water adsorption takes place on two types of adsorption centers: high-energy primary adsorption centers with adsorption values equal to *a*_prim_ and low-energy secondary centers with adsorption values equal to *a*_sec_. As discussed previously, this model describes well the adsorption isotherms of different solids, including carbon adsorbents.^56−58^ The simplified form applied for the description of water adsorption on carbonaceous materials is, in fact, the sum of the Langmuir and Dubinin–Serpinski isotherms^59^
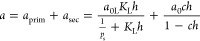
2where *a*_0L_ and *K*_L_ denote adsorption on primary (strong) Langmuir-type sites and the Langmuir constant, respectively, *h* = *p*/*p*_s_ is the relative pressure (*p*_s_ is the saturated vapor pressure, equal to water at 30 °C, 4.2455 kPa), and *a*_0_ and *c* denote adsorption on secondary adsorption sites, and a constant of the Dubinin–Serpinski model, respectively. The values of the parameters obtained from the fitting of eq 2 to water experimental adsorption data and the determination coefficients (*R*^2^) together with the BET surface areas are given in Table 5.

There is a very good correlation between the model and the experimental data. It can be concluded that after annealing in an air atmosphere, increasing adsorption on both types of sites occurs, and the most drastic increase is observed for the 1B-CNOs-a sample (the same effect was also observed after repeated measurements, Figure 6D). To show the quality of the fit and the contribution of adsorption values on both types of sites to the global adsorption isotherm, the data are collected in Figure 6.

In addition to the mentioned sample (1B-CNOs-a), there is a good correlation between the total number of heteroatoms (O + B + N) on the B-CNO surface determined by XPS and the concentration of primary (strong) adsorption sites *a*_0L_ (Figure S7). The unusual adsorption behavior of 1B-CNOs-a results from the formation of hygroscopic boron oxide species during annealing in an air atmosphere. In the presence of moisture, B_2_O_3_ converts to boric acid.^60,61^ The presence of crystalline boric acid was observed on the XRD profile for the 1B-CNOs-a sample (Figure 5).

### 3.3 Nature of Lewis Acid Sites—Quantitative IR Studies of Py Adsorption

**Figure 7:**
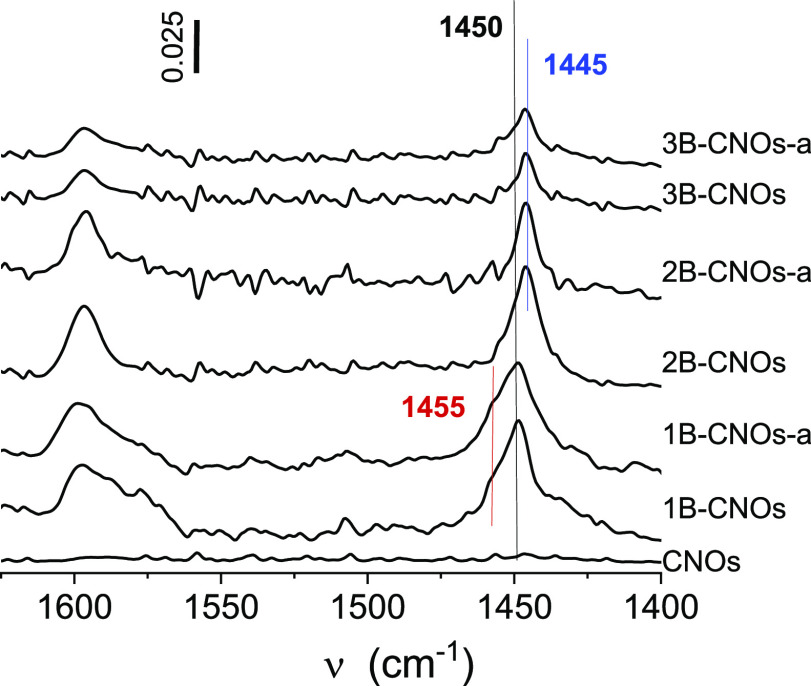
Fourier transform IR spectra of Py adsorbed at 45 °C on the B-doped CNO materials.

The Py molecule is a probe particularly useful for the study of the speciation of the surface centers. Its ability to detect both Brønsted and Lewis acidic centers allows for monitoring of the acidity of the solids by identification of the PyH^+^ ions produced by interaction of Py with Brønsted acid sites (1550–1540 cm^–1^ band) and PyL adducts (1460–1445 cm^–1^ band) produced by the binding of Py to Lewis centers through the free electron pair of the nitrogen atom. Further, the strength of the Lewis acid sites (LAS) reflects the strength of the Py–Lewis interactions which the IR spectrum detects as various positions of the PyL band. The interaction of Py with boron-originated LAS is relatively weak thus the Py adsorption was carried out at 45 °C to assure complete covering of the boron centers with the probe. In the next step, the gas phase and the physically adsorbed Py molecules were removed by desorption to assure the stoichiometry of the interaction between the boron sites and the probe was 1:1. The completeness of the process was documented by the absence of Py bands in the range 1440–1430 cm^–1^. In the spectra of Py interacting with the surface of boron-doped catalysts, the 1460–1445 cm^–1^ bands are present as the only spectral features that indicate the presence of electron-accepting centers (Figure 7). These bands were not found for the boron-free CN material, therefore the appearance of acidic properties (Lewis centers) is undoubtedly related to the doping of CNOs with boron. The concentration of the B-originated centers was calculated using the intensity of the PyL bands and their absorption coefficients (Table 6).^62,63^ The number of Lewis centers strictly reflects the boron content in the CNO’s structure: the higher concentration of Lewis centers was determined for the materials with the higher number of boron atoms introduced. There is a correlation between the Lewis acid site content and the concentration of boron in the carbon matrix in the form of different B carbide species determined by XPS (Figure S8). The annealed materials are characterized by a 10% lower number of acid centers than the native samples due to extraction of boron species from the CNOs matrix and formation of the clustered B_2_O_3_ species that are less accessible to reagents. The effectiveness of the formulation of LAS by boron atoms is reflected in the number of Lewis centers expressed per m^2^. Again, the dispersion of boron centers is best for samples with the highest B content, that is, 1B-CNO and 1B-CNOs-a, in spite of the lowest values of the external surface areas for these materials. The number of boron centers able to bind Py (and thus the reactants) is 4.3-fold higher than for samples with higher surface areas.

**Table 6:** Concentration of LAS Derived from Quantitative IR Studies of Py Adsorption

sample	LAS (μmol/g)	LAS (μmol/m^2^)
CNOs	0	0
1B-CNOs	193	0.94
2B-CNOs	133	0.53
3B-CNOs	64	0.22
1B-CNOs-a	180	0.82
2B-CNOs-a	110	0.42
3B-CNOs-a	70	0.24

The strength of the Lewis centers, thus their nature, is also influenced by the B content (Figure 7). While in 3B-CNOs and 2B-CNOs and their annealed counterparts (3B-CNOs-a and 2B-CNOs-a), only the sites of weak (1445 cm^–1^) and moderate strength are identified (1450 cm^–1^). The nature of the Lewis centers is heterogeneous in the 1B-CNO and 1B-CNO-a materials: besides, the centers of moderate strength (1450 cm^–1^) the centers of significantly higher electron acceptor properties appeared (1455 cm^–1^). The boron-induced Lewis acidity varies: the more B is in the structure, the stronger the acidities are. The IR results correlate well with other spectroscopic experimental results (MAS NMR and XPS) and water adsorption data. The weaker Lewis acidic sites, represented by the band at 1445 cm^–1^, are trigonal boron sites exposed on the B_2_O_3_ surface. The medium and strongly electron accepting Lewis species, detected at 1450 and 1455 cm^–1^, respectively, originate from two types of isolated trigonal boron moieties located in the CNO matrix as the local defects. The boron atoms get electrons from their carbon neighbors, generating charge depletion in their vicinity. Consequently, the electron acceptor properties depend on local electronic structures surrounding the carbon and nitrogen atoms (the formation of boron nitrides was documented by XPS studies). Further, the differentiated Lewis properties of the B-CNOs can arise from the presence of two vicinal boron atoms, that is, B–C–B moieties.^64^ Our IR investigations conclusively showed that in B-CNO materials, there are at least three nonequivalent boron sites.

### 3.4 Catalytic Activity of B-CNOs

The catalytic oxidation of 1.65 × 10^–3^ M aqueous solution of SO_2_ was performed. At this solution concentration, SO_2_ is present as HSO_3_^–^ ions, which may be oxidized by O_2_ to SO_4_^2–^ and H_3_O^+^.^26,27,65^ Due to the low concentrations of all species, and considerably higher the molar conductivity of H_3_O^+^ than those of the rest of the ions, the conversion of SO_2_ can be directly related to the change in the solution conductivity. After adding the catalyst to the solution, its conductivity increased linearly with time until a plateau was reached, when most of the HSO_3_^–^ ions in solution was exhausted. From the slope of the linear part of the curves, the initial catalytic activity was calculated.^26,27,65^

**Figure 8:**
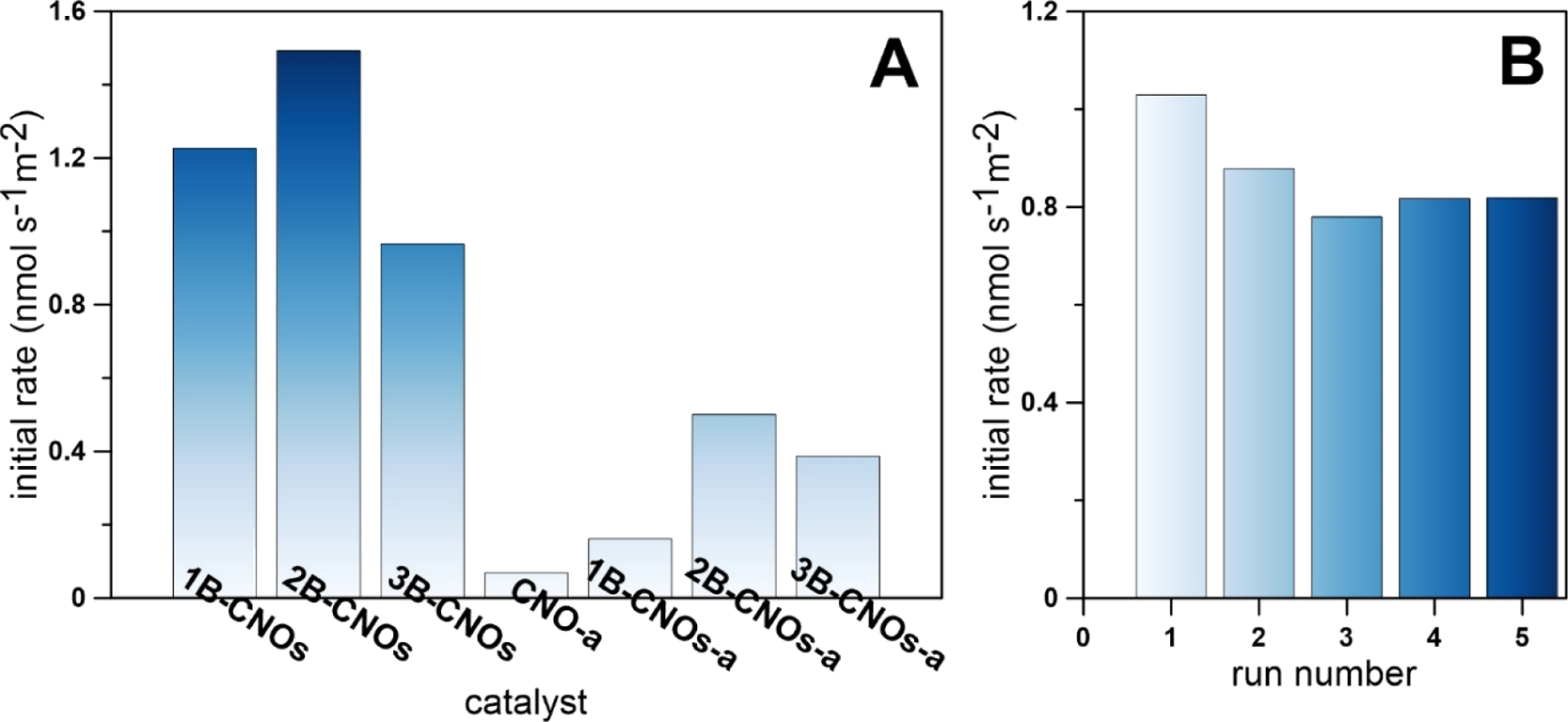
(A) Catalytic activity of B-doped CNOs in SO_2_ oxidation. (B) Catalytic stability of 3B-CNOs in the tests of SO_2_ oxidation.

As expected, all B-CNOs demonstrated quite significant catalytic activity which are higher than those of undoped CNOs-a (Figure 8A). However, after subsequent annealing in an air atmosphere, the B-CNOs catalysts exhibited considerably lower rates of analyte oxidation. The highest rate of SO_2_ oxidation was found for 2B-CNOs. All B-CNO materials are catalytically stable as illustrated in Figure 8B. Unfortunately, no direct relationship between the catalytic activity and the number of heteroatoms on the B-CNO surface was found.

However, it should be noted, that the heteroatom’s synergetic effect depends on the relative location of the heteroatoms rather than their concentration.^28,66−69^ Simultaneous presence of B and N atoms in the CNs, may result in formation of different bonding configurations. For example, B and N atoms can form B–C bonds and N–C bonds, or B atoms can be bonded to N atoms, forming B–N species in the carbon lattice.^28,66−69^ In the case of isolated B and N in carbon matrix, the coupling of extra electron of N and the empty orbital of B to carbon system changes the distribution of electron density, forming charged catalytically active sites.^28,66−70^ Whereas, after the interaction between the extra electron of N atom and the vacant orbital of B atom in bonded B–N, chemisorption of O_2_ become unfavorable.^66,69^ According to the XPS results (Figure 2D, Table 2), the bonded B and N species prevail in the samples, therefore the strong synergetic effect due to B and N co-doping seems to be unlikely. On the other side, we suggest that the high degree of defects in the catalysts (according to Raman analyses) may result in good activity of all B-CNOs. The catalytic activity of the doped CNs affect their ability to disrupt the integrity of π coupling in the graphitic matrix. As reported in the literature, the presence of defects in sp^2^-hybridized C structures may act in similar way.^8,9,71,72^

There is a correlation between the catalytic rate of HSO_3_^–^ oxidation and the Raman reactivity factors (*A*_D3_/*A*_G_, *A*_D3_/*A*_G+D3_, *A*_D3+D4_/*A*_G_) (Figure S9), which characterizes the reactivity of carbon materials with oxygen.^41,43,48,49^ We observed that a higher value of the ratio of these parameters indicates a higher catalytic activity of the doped materials. There is a dependence between the catalytic activity and reactivity factors for each B-CNO series (non-annealed and annealed in an air atmosphere, respectively).

The initial SO_2_ oxidation reaction rate stays constant and independent of density and type of LAS for both the series of native and annealed materials (Figure S10). The catalytic activity decreased after annealing, but there were no differences between the materials with different B-content. The remarkable changes in the number of LAS influenced the SO_2_ oxidation reaction rates to only a small extent. On the other hand, annealing dramatically inhibited SO_2_ oxidation despite the marginal reduction of the number of Lewis centers. This behavior suggests that the ability of the B-doped materials to oxidize HSO_3_^–^ ions is more negatively affected by the segregation of B_2_O_3_ on the catalysts surface (MAS NMR studies) than by the decrease in the total number of Lewis sites. In the presence of water, B_2_O_3_ can be transformed into weak boric acid and therefore affect negatively the transformation of SO_2_ to HSO_3_^–^ and the subsequent oxidation of the latter.

The catalytic performance of carbon materials in oxidation reactions can be attributed to the creation of reactive oxygen forms (ROS) (e.g., ^•^OH, ^•^HO_2_, O_2_^•–^).^65,73^ B doping results in the presence of defects and a high relation of the edge plane surface to the basal plane surface in the graphitic network,^21,74^ which participates in the process of oxygen adsorption and activation on account of the higher electron density than the bulk.^73,75^ In addition, the presence of some incorporated separately B and/or N atoms may affect the band gap of the nanostructure, inducing higher electron mobility and a lower work function at the B-CNO/gas or liquid interface in comparison with the undoped CNOs.^74^ Consequently, the carbon reductive capability for the oxygen adsorption increases, resulting in ROS creation (e.g., ^•^OH, ^•^HO_2_, O_2_^•–^) with a higher oxidation activity than molecular oxygen.^65,73^

The lower catalytic activity of the annealed samples of B-CNO-a, despite higher values of the Raman parameters, can be explained by an inhibition effect of B_2_O_3_ species formed during heating in an air atmosphere.^76^ The lowest activity shown by the 1B-CNO-a sample is a likely result of the highest water adsorption due to the presence of hygroscopic B_2_O_3_ species.

**Figure 9:**
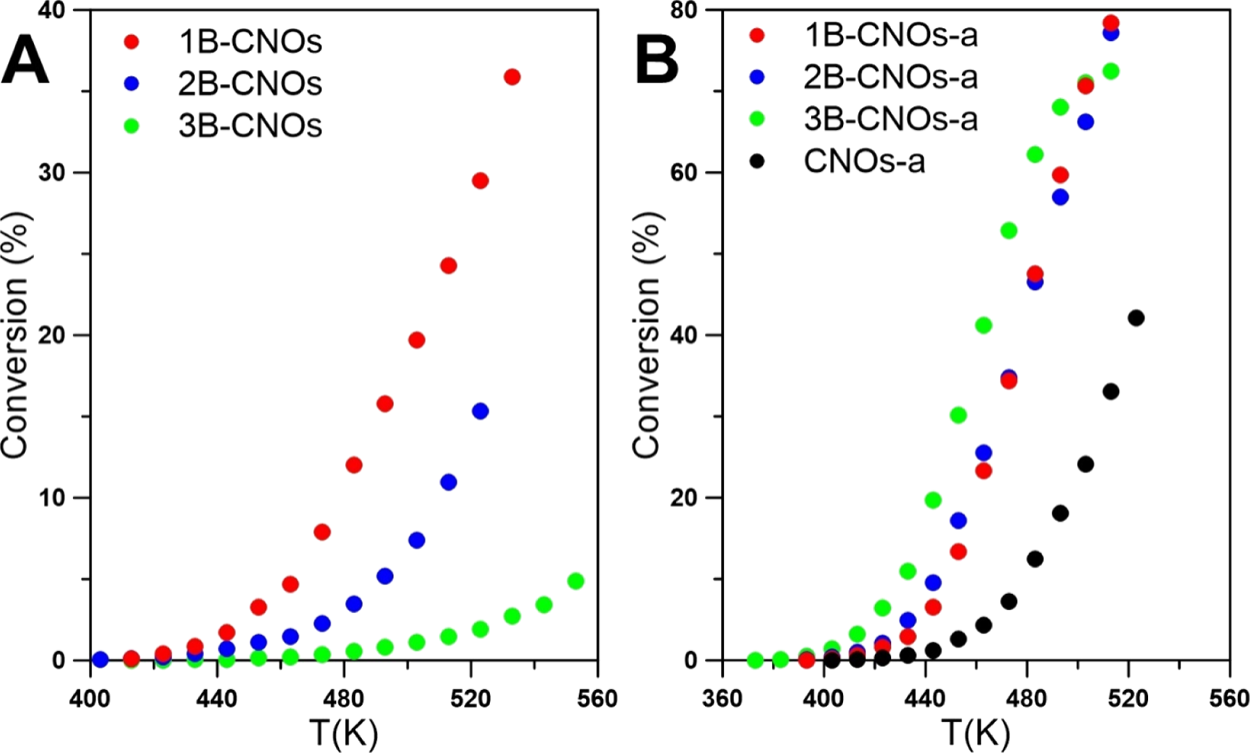
Catalytic activity of the studied B-CNOs in *tert*-butanol dehydration, (A) not annealed; and (B) annealed.

All B-CNO materials, as well as their annealed forms (B-CNOs-a), show catalytic activity for the dehydration reaction of *tert*-butanol (Figure 9). The dehydration activity of CNOs decreases in subsequent runs but it stabilizes after the third cycle (Figure S11). In contrast to SO_2_ oxidation, the most active samples are the B-CNO-a samples, which were annealed in an air atmosphere (Figure 9). On the other hand, the unannealed B-CNOs demonstrate lower activity than undoped CNOs-a. The different activities of both series result from the presence of various acidic sites and/or their different distributions.^84,85^

**Figure 10:**
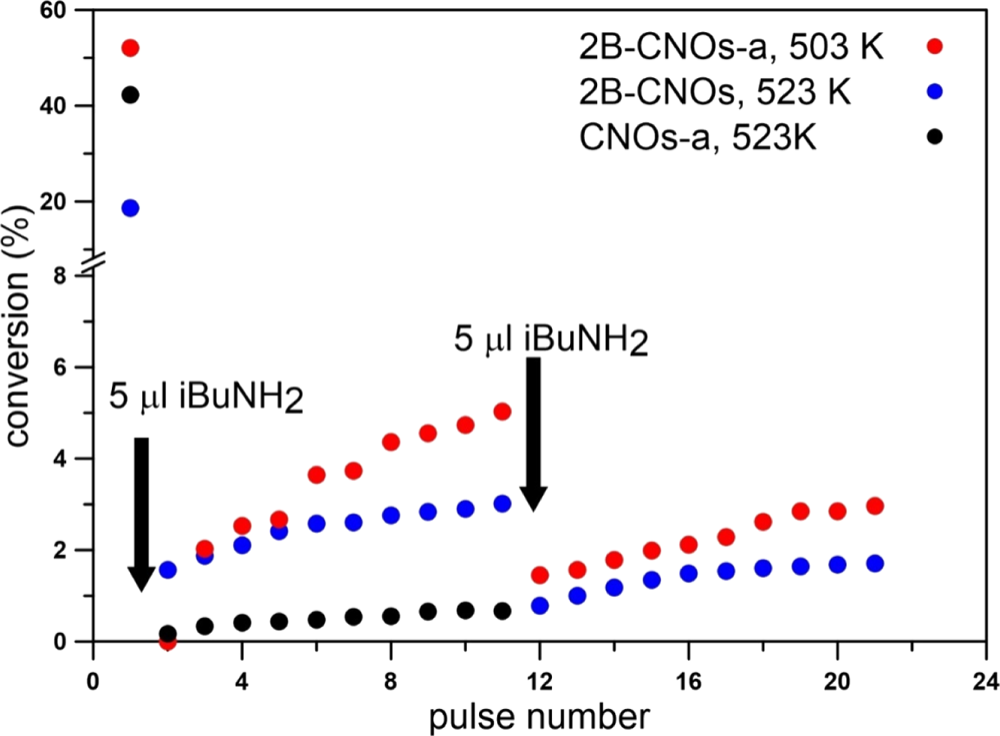
Effect of iso-butylamine (iBuNH_2_) dosing into the microreactor on isobutene formation during subsequent periodic injections of *tert*-butanol (1 μL).

Poisoning of CNOs by injection of iso-butylamine (iBuNH_2_) into the reactor prior to the catalytic tests remarkably deactivated them (Figure 10). The amine interacts more strongly with the acidic sites of undoped CNOs than with the B-doped CNOs samples. As LAS were not detected on the surface of undoped CNOs, the dehydration activity results from the Brønsted acidity, mainly due the presence of carboxylic groups.^77,78^ These groups on the CNO samples can be decomposed at the temperatures applied during sample desorption prior to the IR measurements of Py adsorption and therefore no PyH^+^ bands were observed. The amine groups can chemisorb on the Brønsted acid sites, as well as on the Lewis sites. Therefore, the dehydration activity of the B-doped CNOs can be caused by both types of acidity.

The role of LAS on the catalytic dehydration of *tert*-butanol is clearly illustrated in Figure S12 showing the conversion of *tert*-butanol at 483 and 503 K as a function of LAS density. The linear dependence is observed for native samples evidencing that the higher number of Lewis sites facilitates the dehydration process. The annealing treatment generates a positive effect: the most prominent effects occur for the material with the lowest B-content and all the annealed materials exhibited similar catalytic activity at higher temperatures. Because only isobutene is observed as the sole dehydration reaction product, the pronounced activity of air-heated materials undoubtedly arises from the specific boron sites formed during annealing. The catalytic activity is not importantly differentiated among the samples of various B-content, while such variations are detected for non-annealed materials. The most active is the only material exhibiting an increase of the Lewis site density after air-heating. As documented earlier, the concentration of the oxidized B forms (B_2_O_3_) increased after annealing in an air atmosphere as a result of oxidation of some B and B carbide species. These B_2_O_3_ species react slowly with water to form boric acid which produces hydronium ions that catalyze the transformation of *tert*-butanol to isobutene.

## 4 Conclusions

Boron doping was achieved by obtaining CNOs from ND in the presence of amorphous boron in a high-temperature heating process. The CN doping process was confirmed using direct and indirect measurement methods, such as XPS studies and Raman spectroscopy. All B-CNOs exhibit quite similar elemental surface compositions in spite of different starting ratios of materials: amorphous B to NDs. All samples besides B also contain some amount of O and N on the surface. Undoped CNOs show catalytic and electrocatalytic properties due to numerous surface defects on the nanostructures. Doping with heteroatoms enhances this effect. In this study, we establish that there is a close relationship between the catalytic properties of the B-CNOs and the experimental conditions for their formation. It is not only the mass of the substrates used for the formation of B-CNOs that is crucial, that is, the mass ratio of NDs to amorphous B, but also the process, including temperature and gas atmosphere. As expected, all B-CNOs demonstrated significant catalytic activity in HSO_3_^–^ oxidation. However, the subsequent annealing in an air atmosphere diminished their catalytic activity. Unfortunately, no direct relationship between the catalytic activity and the presence of heteroatoms on the B-CNO surface was observed. There was a dependence between catalytic activity and the Raman reactivity factors for each of the B-CNO materials. The lower catalytic activity of the B-CNO-a samples, despite higher values of the Raman parameters, can be explained on the basis of an inhibition effect of B_2_O_3_ species formed during heating in an air atmosphere. In contrast to SO_2_ oxidation, the B-CNO-a samples showed higher catalytic activity in *tert*-butanol dehydration due to the presence of Lewis acid sites. The most active are the B-CNO-a samples, where the number of oxidized B forms (B_2_O_3_) increased after annealing in an air atmosphere. These B_2_O_3_ species react slowly with water to form boric acid, which produces hydronium ions that catalyze the transformation of *tert*-butanol to isobutene. Our studies clearly showed that the character and number of defective sites introduced into CNOs is crucial for their catalytic activity and may enhance their properties toward analytes. Defects modify the electronic structure of CNOs and optimize the chemisorption of the key intermediates, which should improve catalytic and electrocatalytic performance.

## Supporting Information Available

HRTEM images with EDS analysis of B-CNOs, quadrupolar deconvolution of the central signals and the first order spinning sidebands of 1B-CNOs-a and 2B-CNOs-a, pore—size distributions of B-CNOs calculated using the Barrett–Joyner–Halenda model, correlation between the XPS results and adsorption on Langmuir-type sites, dependence of the LAS density on the concentration of boron carbide-like species determined by the XPS studies, dependence of catalytic activity of B-doped CNOs on the correlation of Raman reactivity parameters for SO_2_ oxidation, dependence of initial rate of SO_2_ oxidation on the LAS density, catalytic stability of B-CNOs in the tests of *tert*-butanol dehydration, dependence of *tert*-butanol conversion on the LAS density, XPS and Raman analyses of B-CNOs (PDF)
