# Supplementary material for: Linking the Defective Structure of Boron-Doped Carbon Nano-Onions with Their Catalytic Properties: Experimental and Theoretical Studies
Source: ACS Appl Mater Interfaces. 2021 Oct 22;13(43):51628–42. doi: 10.1021/acsami.1c12126 (PMC8569677; doi:10.1021/acsami.1c12126)
Supplement: Supplementary file 1 — am1c12126_si_001.pdf [file am1c12126_si_001.pdf]

Supporting Information

Linking the Defective Structure of Boron-Doped Carbon Nano-Onions with Their Catalytic Properties: Experimental and Theoretical Studies

Grzegorz S. Szymański,[1] Yuka Suzuki[2], Tomonori Ohba[2], Bogdan Sulikowski,[3] Kinga Góra-Marek[4], Karolina A. Tarach[4], Stanisław Koter,[5] Piotr Kowalczyk[6], Anna Ilnicka[7], Monika Zięba[1], Luis Echegoyen, [8] Artur P. Terzyk,[1]* and Marta E. Plonska-Brzezinska[9]**

[1] Faculty of Chemistry, Physicochemistry of Carbon Materials Research Group, Nicolaus Copernicus University in Torun, Gagarin Street 7, 87-100 Torun, Poland
[2] Graduate School of Science, Chiba University, 1-33 Yayoi, Inage, Chiba 263-8522, Japan
[3] Jerzy Haber Institute of Catalysis and Surface Chemistry, Polish Academy of Science, Niezapominajek 8, 30-239 Cracow, Poland
[4] Faculty of Chemistry, Jagiellonian University in Kraków, Gronostajowa Street 2, 30-387 Kraków, Poland
[5] Faculty of Chemistry, Department of Physical Chemistry, Nicolaus Copernicus University in Torun, Gagarin Street 7, 87-100 Torun, Poland
[6] School of Engineering and Information Technology, Murdoch University, Murdoch 6150 Western Australia, Australia
[7] Faculty of Chemistry, Nicolaus Copernicus University in Torun, Gagarin Street 7, 87-100 Torun, Poland
[8] Department of Chemistry, University of Texas at El Paso, 500 W. University Ave., El Paso, TX 79968 USA
[9] Department of Organic Chemistry, Faculty of Pharmacy with the Division of Laboratory Medicine, Medical University of Bialystok, Mickiewicza 2A, 15-222 Bialystok, Poland

Correspondence: aterzyk@chem.uni.torun.pl (A.P.T.); marta.plonska-brzezinska@umb.edu.pl (M.E.P-B.)

Figure S1. (A, C) HRTEM images with (B, D) EDS analysis of the (A, B) 1B-CNOs and (C, D) 1B-CNOs-a.

Figure S2. (A, C) HRTEM images with (B, D) EDS analysis of the (A, B) 2B-CNOs and (C, D) 2B-CNOs-a.

Figure S3. (A, C) HRTEM images with (B, D) EDS analysis of the (A, B) 3B-CNOs and (C, D) 3B-CNOs-a.

Figure S4. Quadrupolar deconvolution of the central signals and the first order spinning sidebands of the 1B-CNOs-a sample. The simulated spectrum is marked in red. Correlation coefficient R = 96.47 % (cf. Table 3).

Figure S5. Quadrupolar deconvolution of the central signals and the first order spinning sidebands of the 2B-CNOs-a sample. The simulated spectrum is marked in red. Correlation coefficient R = 96.85 % (cf. Table 3).

Figure S6. Pore – size distributions of studied B-CNOs calculated using the BJH model with KJS corrections.

Figure S7. Correlation between the XPS results and adsorption on primary (strong) Langmuir-type sites calculated using the D'Arcy and Watt model.

Figure S8. The dependence of the Lewis acid sites density on the concentration of boron carbide-like species determined by the FTIR studies.

Figure S9. The dependence of catalytic activity of B-doped CNOs on the correlation of Raman reactivity parameters for SO_2 oxidation.

Figure S10. The dependence of initial rate of SO_2 oxidation on the Lewis acid sites density.

Figure S11. Catalytic stability of the studied B-CNOs in the tests of *tert*-butanol dehydration. (A) CNOs-a, (B) 3B-CNOs-a and (C) 1B-CNOs.

Figure S12. The dependence of *tert*-butanol conversion on the Lewis acid sites density.

Table S1. Chemical state, positions, FWHM and relative area percentages of the deconvoluted C1s peaks obtained from XPS analyses of B-CNOs.

Table S2. Chemical state, positions, FWHM and relative area percentages of the deconvoluted C1s peaks obtained from XPS analyses of B-CNOs.

Table S3. Chemical state, positions, FWHM and relative area percentage of the deconvoluted B1s and N1s peaks obtained from XPS analyses of B-CNOs.

Table S4. Parameters of fitted first-order Raman spectra.

Table S5. Parameters of fitted second-order Raman spectra.

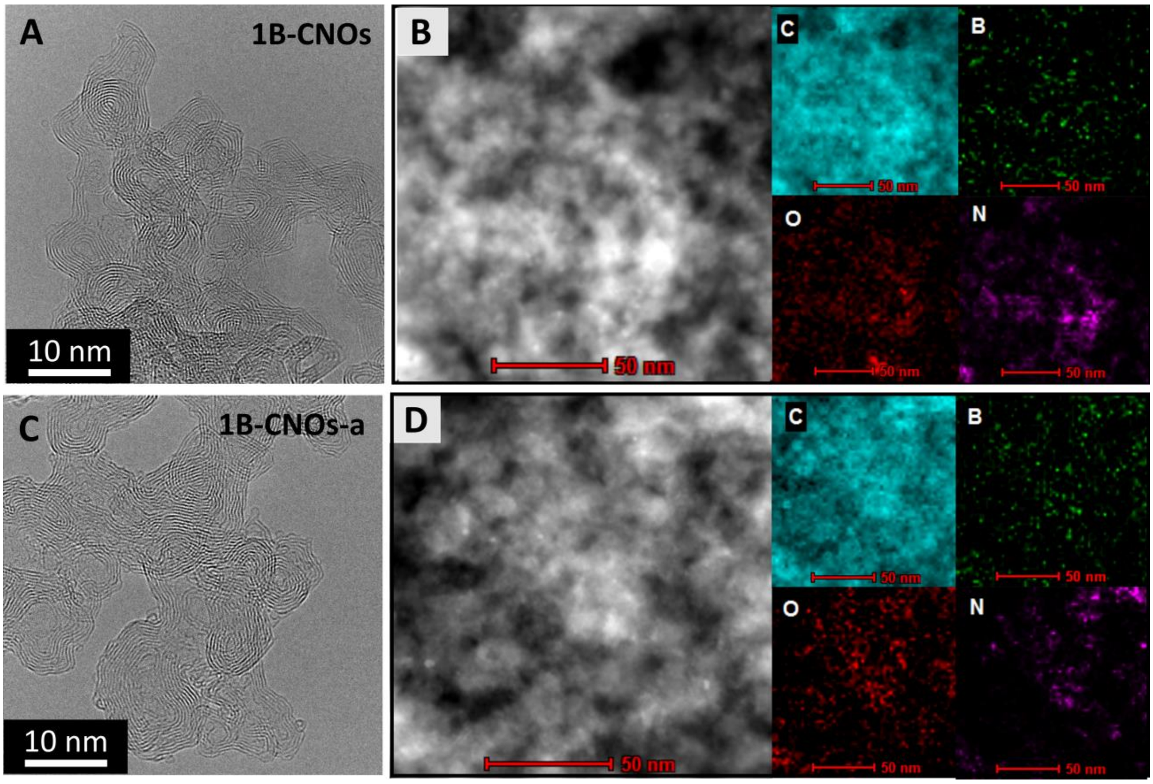

Figure S1. (A, C) HRTEM images with (B, D) EDS analysis of the (A, B) 1B-CNOs and (C, D) 1B-CNOs-a.

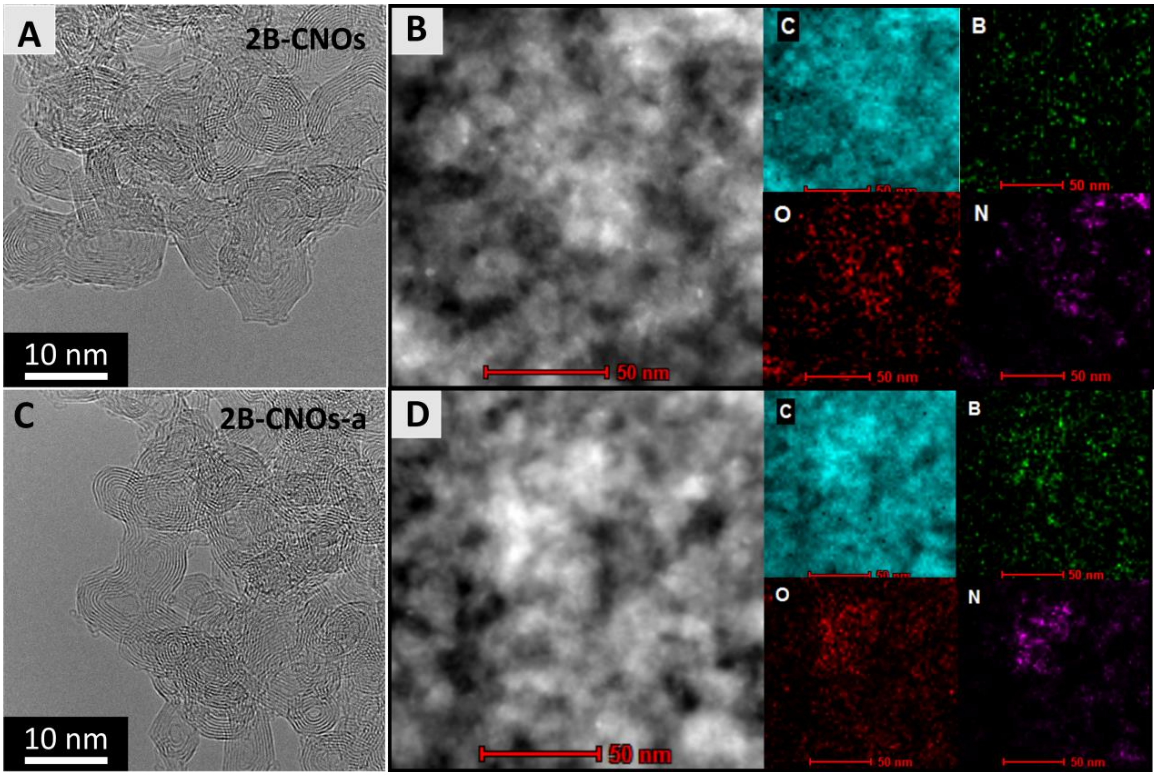

Figure S2. (A, C) HRTEM images with (B, D) EDS analysis of the (A, B) 2B-CNOs and (C, D) 2B-CNOs-a.

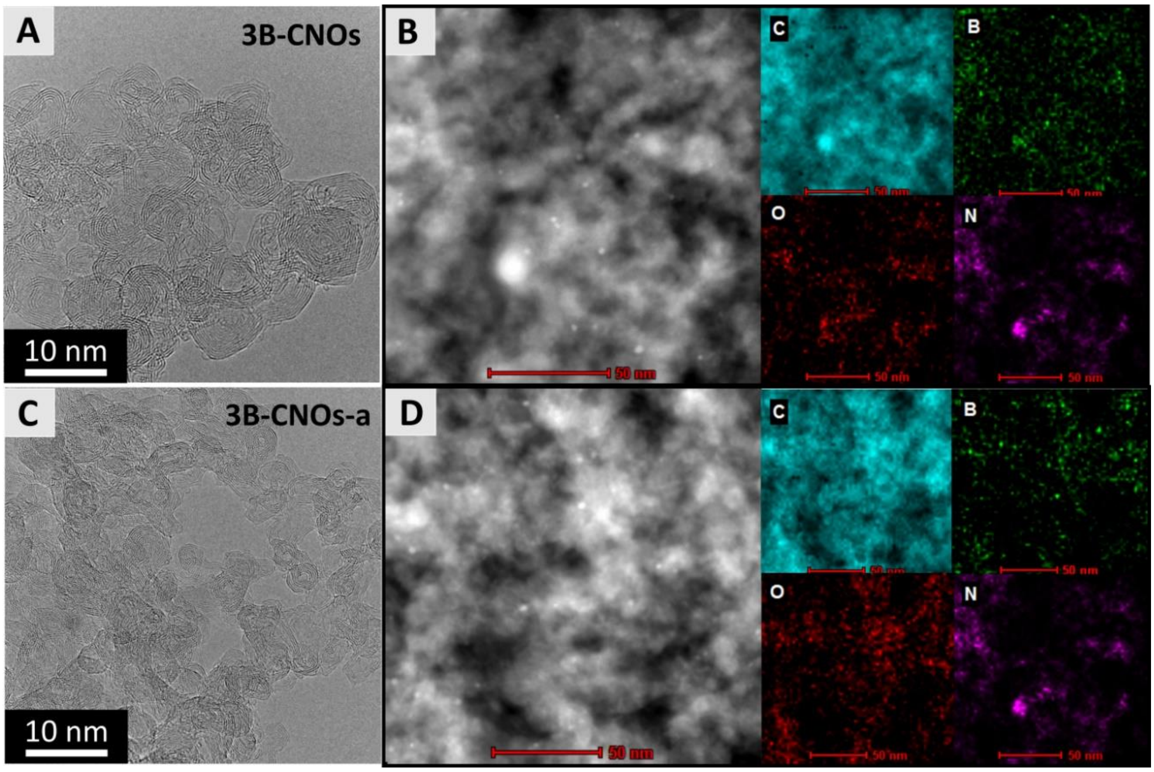

Figure S3. (A, C) HRTEM images with (B, D) EDS analysis of the (A, B) 3B-CNOs and (C, D) 3B-CNOs-a.

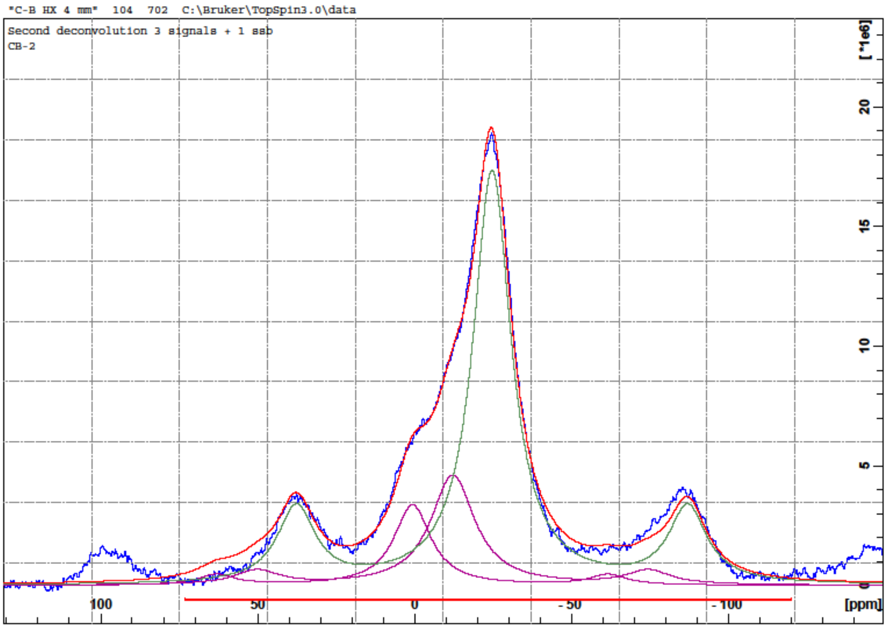

Figure S4. Quadrupolar deconvolution of the central signals and the first order spinning sidebands of the 1B-CNOs-a sample. The simulated spectrum is marked in red. Correlation coefficient R = 96.47 % (cf. Table 3).

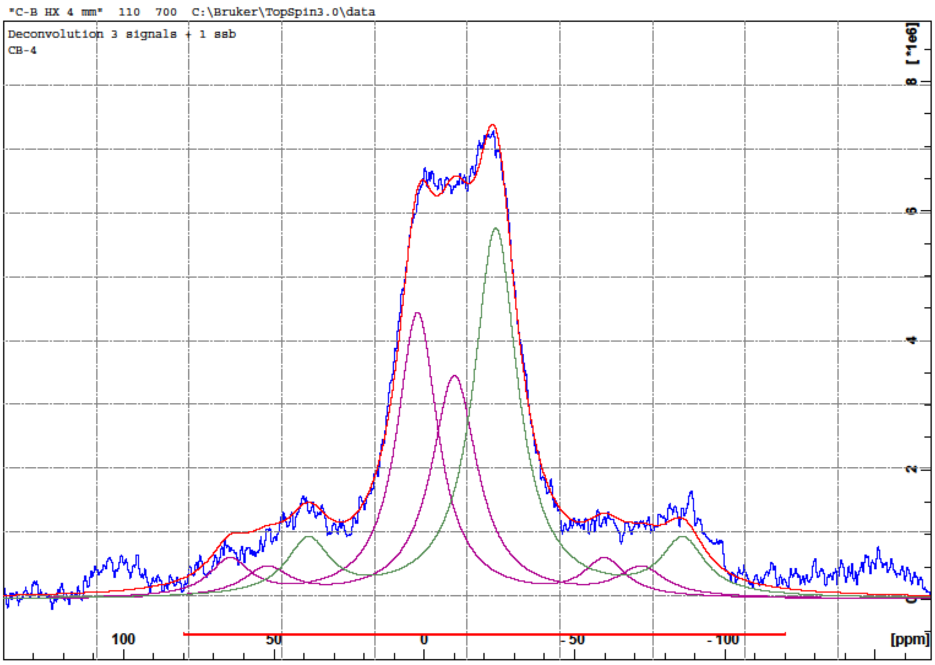

Figure S5. Quadrupolar deconvolution of the central signals and the first order spinning sidebands of the 2B-CNOs-a sample. The simulated spectrum is marked in red. Correlation coefficient R = 96.85 % (cf. Table 3).

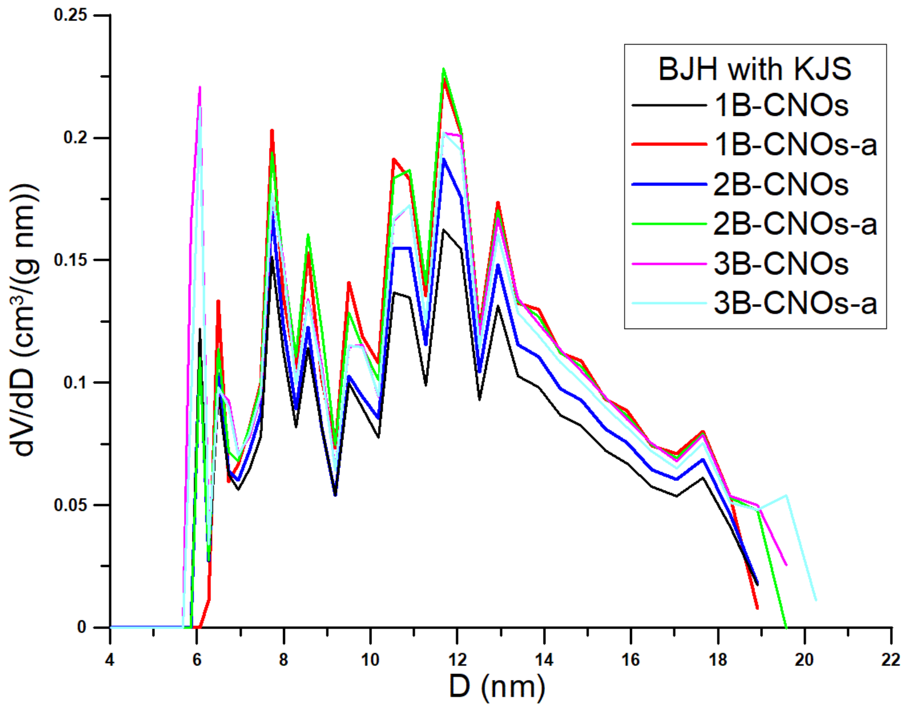

Figure S6. Pore – size distributions of studied B-CNOs calculated using the BJH model with KJS corrections.

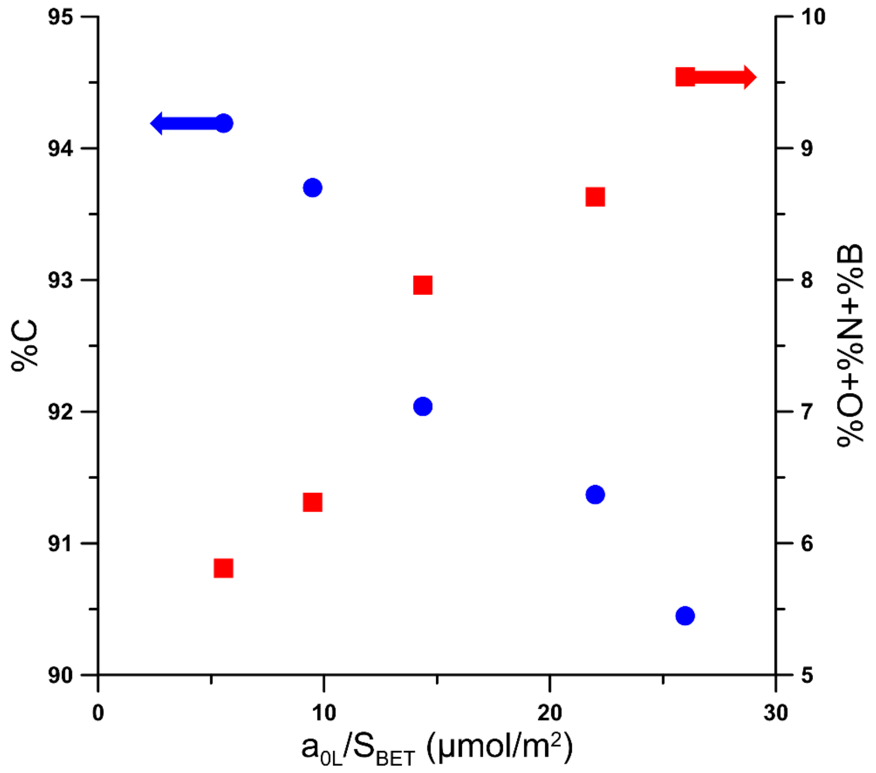

Figure S7. Correlation between the XPS results and adsorption on primary (strong) Langmuir-type sites calculated using the D'Arcy and Watt model.

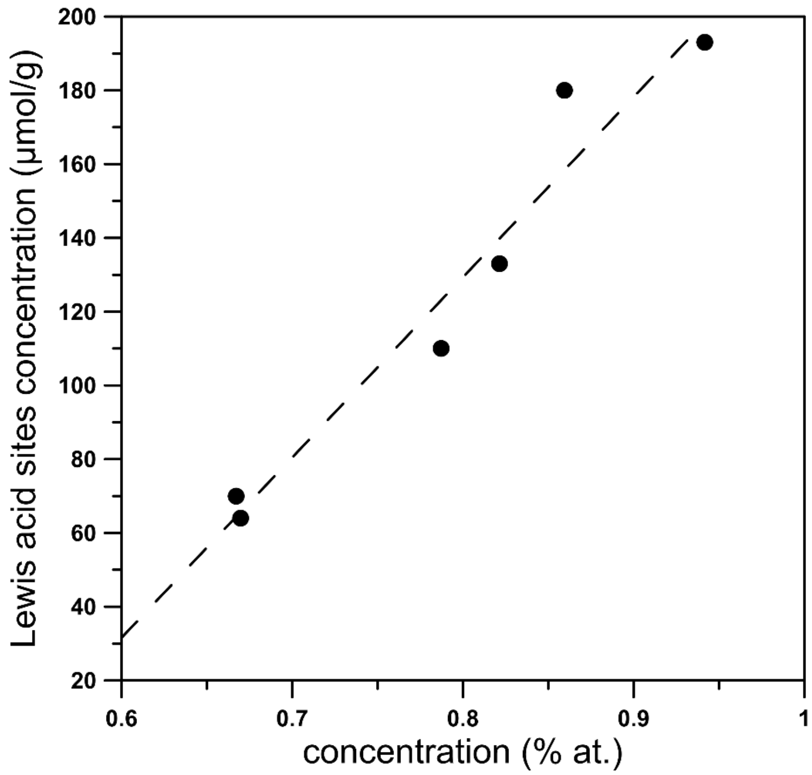

Figure S8. The dependence of the Lewis acid sites density on the concentration of boron carbide-like species determined by the FTIR studies.

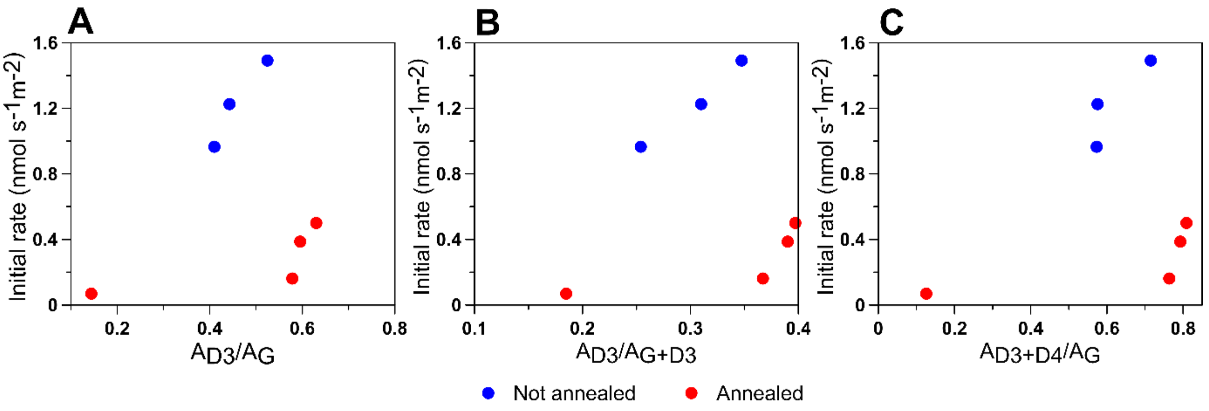

Figure S9. The dependence of catalytic activity of B-doped CNOs on the correlation of Raman reactivity parameters for SO_2 oxidation.

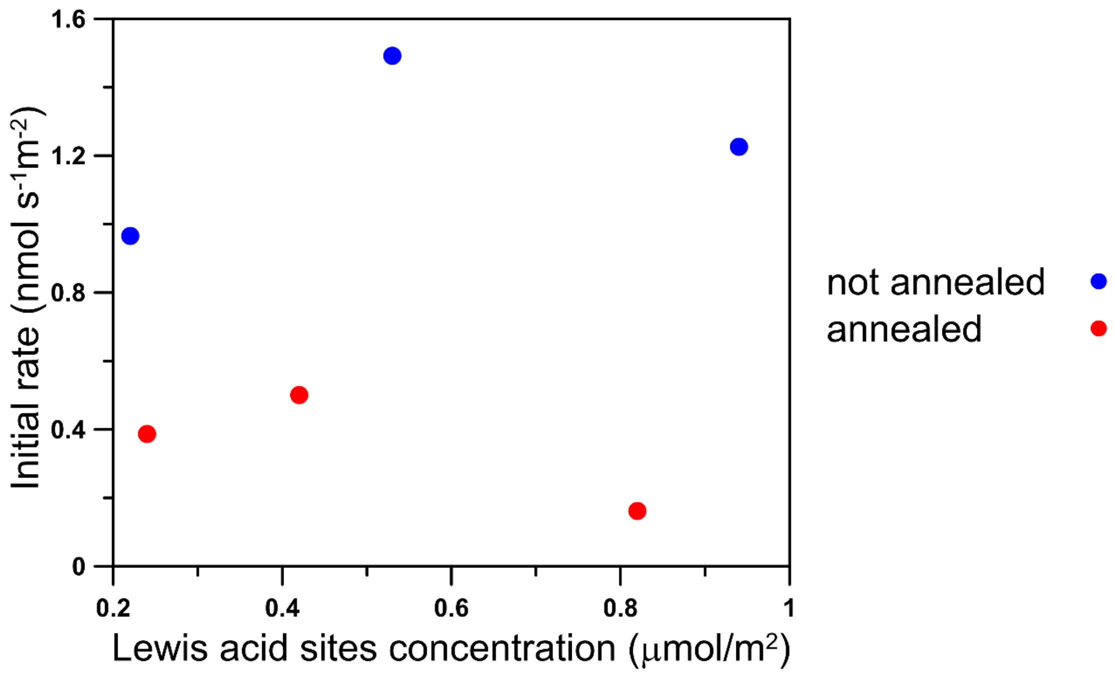

Figure S10. The dependence of initial rate of SO_2 oxidation on the Lewis acid sites density.

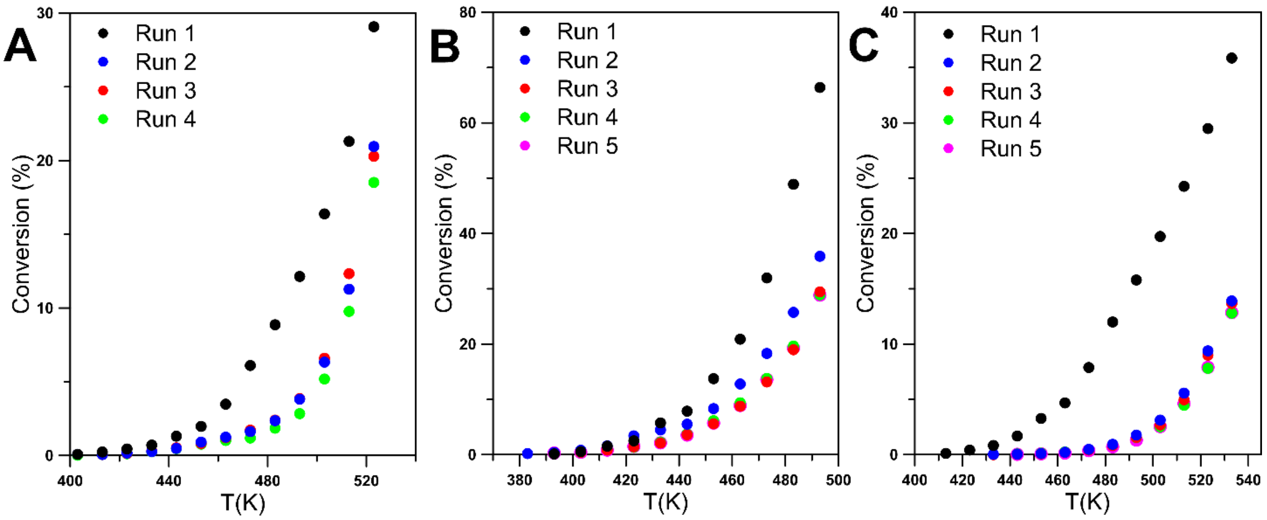

Figure S11. Catalytic stability of the studied B-CNOs in the tests of *tert*-butanol dehydration, (A) CNOs-a, (B) 3B-CNOs-a and (C) 1B-CNOs.

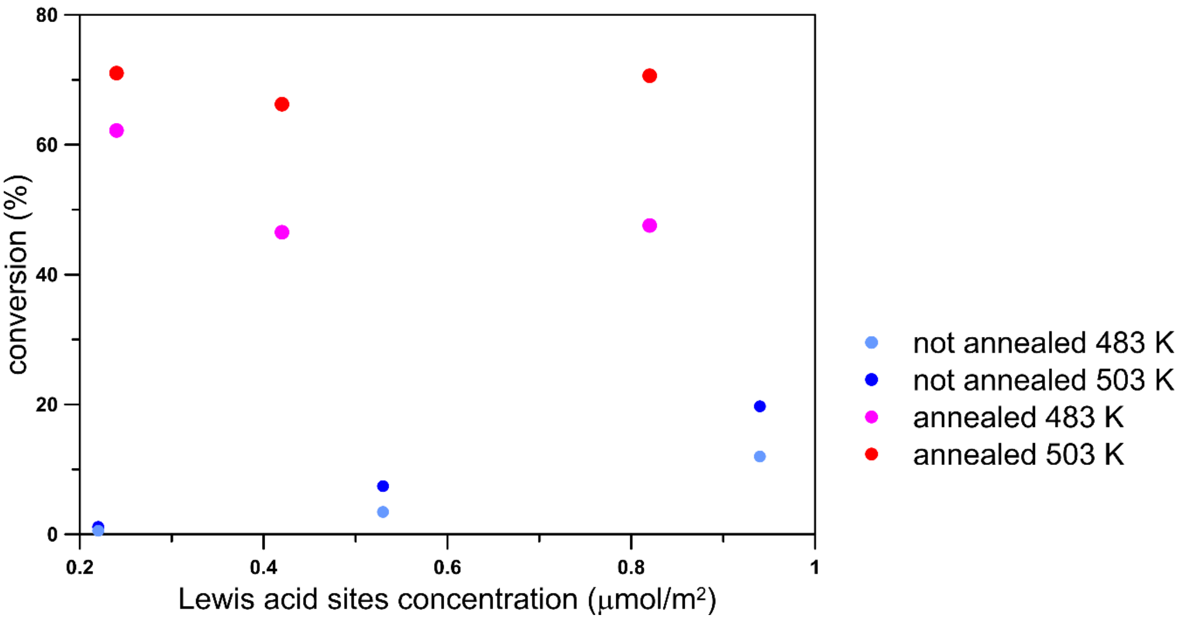

Figure S12. The dependence of *tert*-butanol conversion on the Lewis acid sites density.

Table S1. Chemical state, positions, FWHM and relative area percentages of the deconvoluted C1s peaks obtained from XPS analyses of B-CNOs

Sample	Parameters	Peak/Species								
		A	B	C	D	E	F	G	H	I
		C-C sp^2	Strained C-C sp^2	C-C sp^3	C-OH	C-O-C	C=O	O=C-O	C-B	$\pi-\pi^*$
**1B-CNOs**	Peak (eV)	284.11	284.51	285.11	285.72	286.38	287.13	288.69	282.57	290.24
	FWHM (eV)	0.72	0.78	0.78	0.76	0.71	0.82	0.54	0.85	1.61
	%Area	89.6	0.7	2.7	2.7	1.6	0.8	0.2	1.7	-
**2B-CNOs**	Peak (eV)	284.18	284.51	285.21	285.77	286.41	287.00	288.68	282.67	290.11
	FWHM (eV)	0.72	0.78	0.78	0.78	0.77	0.84	0.48	0.85	1.82
	%Area	90.2	0.5	3.1	2.5	1.2	0.6	0.2	1.7	-
**3B-CNOs**	Peak (eV)	284.18	284.51	285.21	285.77	286.41	287.00	288.68	282.67	290.15
	FWHM (eV)	0.70	0.76	0.76	0.76	0.75	0.83	0.47	0.83	1.71
	%Area	91.8	0.3	2.8	2.2	1.0	0.7	0.0	1.2	-
**1B-CNOs-a**	Peak (eV)	284.12	284.51	285.15	285.70	286.31	287.04	288.72	282.56	290.33
	FWHM (eV)	0.73	0.80	0.80	0.80	0.79	0.83	0.49	0.87	1.59
	%Area	92.1	0.1	1.9	2.1	1.3	0.6	0.0	2.0	-
**2B-CNOs-a**	Peak (eV)	284.14	284.62	285.13	285.75	286.49	286.91	288.75	282.56	290.15
	FWHM (eV)	0.72	0.78	0.79	0.79	0.77	0.86	0.49	0.85	1.76
	%Area	87.8	2.3	3.9	2.8	1.2	0.3	0.1	1.6	-
**3B-CNOs-a**	Peak (eV)	284.17	284.58	285.17	285.74	286.47	287.21	288.94	282.59	290.2
	FWHM (eV)	0.70	0.77	0.77	0.77	0.76	0.82	0.48	0.83	1.73
	%Area	92.2	0.1	2.0	2.5	1.5	0.3	0.2	1.2	-

Table S2. Chemical state, positions, FWHM and relative area percentages of the deconvoluted C1s peaks obtained from XPS analyses of B-CNOs

**Sample**	**Parameters**	**Peak/Species**			
		A	B	C	D
		O-B	HO-C	C-O-C	H_2O
**1B-CNOs**	Peak (eV)	531.25	532.39	533.32	534.41
	FWHM (eV)	1.32	1.24	1.23	1.46
	%Area	10.0	40.8	35.8	13.4
**2B-CNOs**	Peak (eV)	531.54	532.47	533.44	534.58
	FWHM (eV)	1.3	1.22	1.21	1.44
	%Area	10.6	42.9	38.0	8.6
**3B-CNOs**	Peak (eV)	531.64	532.47	533.38	534.64
	FWHM (eV)	1.31	1.23	sty.22	1.45
	%Area	12.3	38.7	40.6	8.5
**1B-CNOs-a**	Peak (eV)	531.45	532.52	533.36	534.45
	FWHM (eV)	1.35	1.27	1.26	1.49
	%Area	16.3	39.7	34.8	9.2
**2B-CNOs-a**	Peak (eV)	531.53	532.40	533.32	534.43
	FWHM (eV)	1.51	1.42	1.41	1.67
	%Area	12.8	37.2	41.5	8.4
**3B-CNOs-a**	Peak (eV)	531.54	532.53	533.41	534.53
	FWHM (eV)	1.4	1.32	1.31	1.55
	%Area	17.7	42.1	35.1	5.2

Table S3. Chemical state, positions, FWHM and relative area percentage of the deconvoluted B1s and N1s peaks obtained from XPS analyses of B-CNOs.

Sample	Parameters	Peak/Species					
		A	B	C	D	N1s A	N1s B
		B, B_4C	B-C	BC_2O, B-N	B_2O_3	N-B	C-N
**1B-CNOs**	Peak (eV)	187.38	188.55	190.12	192.06	397.82	399.99
	FWHM (eV)	1.31	1.31	1.5	2.54	1.5	1.5
	%Area	23.5	21.0	47.4	8.1	92.0	8.0
**2B-CNOs**	Peak (eV)	187.31	188.48	190.05	192.00	397.76	399.44
	FWHM (eV)	1.41	1.41	1.62	2.74	1.41	1.41
	%Area	22.2	18.3	50.1	9.4	94.7	5.3
**3B-CNOs**	Peak (eV)	187.36	188.71	190.05	192.16	397.71	399.35
	FWHM (eV)	1.27	1.28	1.46	2.48	1.48	1.48
	%Area	25.8	25.7	36.5	12.0	89.1	10.9
**1B-CNOs-a**	Peak (eV)	187.38	188.54	190.12	192.08	397.84	399.56
	FWHM (eV)	1.4	1.39	1.6	2.74	1.49	1.49
	%Area	23.2	20.3	41.7	14.8	91.3	8.7
**2B-CNOs-a**	Peak (eV)	187.34	188.51	190.08	192.03	397.79	399.35
	FWHM (eV)	1.39	1.39	1.6	2.71	1.41	1.41
	%Area	19.7	19.4	47.5	13.4	91.6	8.4
**3B-CNOs-a**	Peak (eV)	187.25	188.40	189.84	192.16	397.66	398.78
	FWHM (eV)	1.41	1.41	1.61	2.74	1.33	1.33
	%Area	22.2	18.4	40.6	18.8	85.0	15.0

Table S4. Parameters of fitted first-order Raman spectra.

Sample	Parameter	Peak			
		D4	D1	D3	G
**1B-CNOs**	Center (cm^{-1})	1130	1346	1525	1589
	Hight (a.u.)	0.028	0.921	0.153	0.679
	FWHM (cm^{-1})	226	85	124	69
	Area (a.u.× cm^{-1})	6.69	117.26	22.35	50.54
**2B-CNOs**	Center (cm^{-1})	1129	1338	1517	1578
	Hight (a.u.)	0.033	0.879	0.158	0.638
	FWHM (cm^{-1})	250	90	130	70
	Area (a.u.× cm^{-1})	9.41	120.64	25.61	48.56
**3B-CNOs**	Center (cm^{-1})	1128	1338	1515	1580
	Hight (a.u.)	0.034	0.900	0.123	0.644
	FWHM (cm^{-1})	213	83	141	70
	Area (a.u.× cm^{-1})	8.27	112.46	20.72	50.79
**1B-CNOs-a**	Center (cm^{-1})	1127	1340	1526	1581
	Hight (a.u.)	0.036	0.898	0.171	0.617
	FWHM (cm^{-1})	225	92	140	69
	Area (a.u.× cm^{-1})	8.54	122.81	26.63	46.27
**2B-CNOs-a**	Center (cm^{-1})	1121	1338	1519	1581
	Hight (a.u.)	0.034	0.903	0.170	0.624
	FWHM(cm^{-1})	214	89	127	69
	Area (a.u.× cm^{-1})	8.22	119.87	28.70	45.85
**3B-CNOs-a**	Center (cm^{-1})	1130	1336	1509	1580
	Hight (a.u.)	0.037	0.881	0.128	0.584
	FWHM (cm^{-1})	221	83	145	69
	Area (a.u.× cm^{-1})	8.74	109.54	26.35	44.31
**CNOS-a**	Center (cm^{-1})	1089.9	1346.3	1524.1	1591.6
	Hight (a.u.)	0.016	0.981	0.111	0.776
	FWHM (cm^{-1})	135.2	76.0	70.4	69.9
	Area (a.u.× cm^{-1})	2.37	107.76	8.35	57.95

Table S5. Parameters of fitted second-order Raman spectra.

Sample	Parameter	Peak				
		D1+D4	2D1	D1+D3	D1+G	2G
**1B-CNOs**	Center (cm^{-1})	2480	2684	2872	2937	3197
	Hight (a.u.)	0.013	0.184	0.018	0.080	0.012
	FWHM (cm^{-1})	195	149	126	140	118
	Area (a.u.× cm^{-1})	2.679	40.225	2.683	16.631	1.451
**2B-CNOs**	Center (cm^{-1})	2512	2670	2838	2918	3173
	Hight (a.u.)	0.025	0.152	0.031	0.068	0.009
	FWHM (cm^{-1})	231	145	220	149	117
	Area (a.u.× cm^{-1})	6.063	28.482	8.792	14.735	1.104
**3B-CNOs**	Center (cm^{-1})	2462	2664	2857	2921	3182
	Hight (a.u.)	0.017	0.227	0.025	0.074	0.012
	FWHM (cm^{-1})	155	131	143	136	114
	Area (a.u.× cm^{-1})	2.780	44.106	4.710	14.804	1.459
**1B-CNOs-a**	Center (cm^{-1})	2498	2675	2831	2924	3170
	Hight (a.u.)	0.021	0.155	0.020	0.081	0.011
	FWHM (cm^{-1})	220	157	140	160	137
	Area (a.u.× cm^{-1})	4.862	33.279	3.317	17.991	1.625
**2B-CNOs-a**	Center (cm^{-1})	2475	2670	2861	2919	3175
	Hight (a.u.)	0.013	0.163	0.015	0.077	0.010
	FWHM(cm^{-1})	173	161	175	154	120
	Area (a.u.× cm^{-1})	2.343	37.831	2.913	17.312	1.318
**3B-CNOs-a**	Center (cm^{-1})	2477	2664	2811	2913	3170
	Hight (a.u.)	0.015	0.181	0.015	0.084	0.012
	FWHM (cm^{-1})	192	123	185	147	126
	Area (a.u.× cm^{-1})	3.039	33.319	3.524	18.264	1.636
**CNOs-a**	Center (cm^{-1})	2467.4	2682.1	-	2944.1	3229.3
	Hight (a.u.)	0.010	0.161	-	0.061	0.014
	FWHM (cm^{-1})	64.2	114	-	107	80.8
	Area (a.u.× cm^{-1})	0.937	24.839	-	7.001	1.683
